# Genome and chromosome wide association studies for growth traits in Simmental and Simbrah cattle

**DOI:** 10.5713/ab.21.0517

**Published:** 2022-06-30

**Authors:** René Calderón-Chagoya, Vicente Eliezer Vega-Murillo, Adriana García-Ruiz, Ángel Ríos-Utrera, Guillermo Martínez-Velázquez, Moisés Montaño-Bermúdez

**Affiliations:** 1 Facultad de Medicina Veterinaria y Zootecnia, Universidad Nacional Autónoma de México, Ciudad Universitaria, Ciudad de México 04510, México; 2 Centro Nacional de Investigación Disciplinaria en Fisiología y Mejoramiento Animal, Instituto Nacional de Investigaciones Forestales, Agrícolas y Pecuarias, Colón, Querétaro 76280, México; 3 Facultad de Medicina Veterinaria y Zootecnia, Universidad Veracruzana, Veracruz, Veracruz 91710, México; 4 Campo Experimental La Posta, Instituto Nacional de Investigaciones Forestales, Agrícolas y Pecuarias, Medellín, Veracruz 94277, México; 5 Campo Experimental Santiago Ixcuintla, Instituto Nacional de Investigaciones Forestales, Agrícolas y Pecuarias, Santiago Ixcuintla, Nayarit 63570, México

**Keywords:** Genes, Growth Traits, Genome Wide Association Studies (GWAS), Simbrah, Simmental, Single Nucleotide Polymorphism (SNP)

## Abstract

**Objective:**

The objective of this study was to perform genome (genome wide association studies [GWAS]) and chromosome (CWAS) wide association analyses to identify single nucleotide polymorphisms (SNPs) associated with growth traits in registered Simmental and Simbrah cattle.

**Methods:**

The phenotypes were deregressed BLUP EBVs for birth weight, weaning weight direct, weaning weight maternal, and yearling weight. The genotyping was performed with the GGP Bovine 150k chip. After the quality control analysis, 105,129 autosomal SNP from 967 animals (473 Simmental and 494 Simbrah) were used to carry out genotype association tests. The two association analyses were performed per breed and using combined information of the two breeds. The SNP associated with growth traits were mapped to their corresponding genes at 100 kb on either side.

**Results:**

A difference in magnitude of posterior probabilities was found across breeds between genome and chromosome wide association analyses. A total of 110, 143, and 302 SNP were associated with GWAS and CWAS for growth traits in the Simmental-, Simbrah- and joint-data analyses, respectively. It stands out from the enrichment analysis of the pathways for RNA polymerase (POLR2G, POLR3E) and GABAergic synapse (GABRR1, GABRR3) for Simmental cattle and p53 signaling pathway (BID, SERPINB5) for Simbrah cattle.

**Conclusion:**

Only 6,265% of the markers associated with growth traits were found using CWAS and GWAS. The associated markers using the CWAS analysis, which were not associated using the GWAS, represents information that due to the model and priors was not associated with the traits.

## INTRODUCTION

Simmental and Simbrah cattle are some of the most widespread breeds for meat production in Mexico. Growth traits are traditionally included as selection criteria in beef cattle breeding programs, due to their association with meat production and therefore are of great economic importance for both breeders and industry [1]. The most common type of growth trait used in the selection process is the body weight measurement, which can be taken at birth and throughout an animal’s life [2]. Growth traits usually present heritability and genetic correlations from moderate to strong [3,4]. Genetic associations are caused by linkage disequilibrium (LD) and pleiotropic effects of genes [5]. The LD is important for experimental designs to increase genome wide association studies (GWAS) efficiency in studied populations. Linkage disequilibrium patterns and scale within and between populations/breeds can be influenced by several factors, such as marker allele frequencies, selection history, population structure, effective population size, marker type and density, and LD measure used [6]. Additionally, inter-chromosome epistasis effects are expected to be unaffected by LD between the two single nucleotide polymorphisms (SNPs) of each SNP pair but some effects still could have contributions from LD between an SNP adjacent to another SNP that had a significant inter-chromosome epistasis effect [7]. The aim of this research was to implement GWAS and chromosome-wide association (CWAS) analyses to identify SNPs associated with growth traits in registered Simmental and Simbrah cattle.

## MATERIALS AND METHODS

The genotype and phenotype of 1,130 animals (547 Simmental and 583 Simbrah), provided by the Mexican Simmental-Simbrah Breeders Association, were used. The phenotypes were deregressed BLUP EBV [8] for birth weight (BW), weaning weight direct (WWD), weaning weight maternal (WWM), and yearling weight (YW).

Blood samples were taken from all the animals in the study. Samples were individually identified and sent to Neogen’s GeneSeek Laboratory (Lincoln, NE, USA) for DNA extraction and genotyping using the GGP Bovine 150k chip with 138,962 SNP.

All SNP with a call rate <0.95 and a minor allele frequency <0.05 were excluded. Individuals with a call rate less than 0.95 were also deleted. After the quality control analysis, 105,129 autosomal SNP from 967 animals (473 Simmental and 494 Simbrah) were used to carry out genotype association tests. Additionally, chromosome marker databases were performed to calculate their effect on the different traits individually. Intra-chromosomal LD were evaluated by means of pair-wise coefficients of determination (r^2^) in Plink 1.9. Marker pairs were grouped based on their pairwise physical distance into bins of 1 kb, starting from 0 to 5,000 kb. The average r^2^ for SNP pairs in each bin was estimated as the arithmetic mean of all r^2^.

A comparison was made between the GWAS and CWAS analyses using a Bayes B model through the BGLR statistical package of the R program. These association analyses were carried out separately for the two breeds and combining the information of both breeds (joint analysis).

For a continuous response (***y****_i_*; i = 1, ..., n) the data equation is represented as ***y****_i_* = ***η****_i_* + ***ɛ****_i_*, where ***η****_i_* is a linear predictor (the expected value of ***y****_i_* given predictors) and ***ɛ****_i_* are the residuals, independent and normally distributed with mean zero and variance $w_{i}^{2}\sigma_{ɛ}^{2}$. The linear predictor represents the conditional expectation function, and it is structured as


$$\eta =1\mu +\sum_{j=1}^{J}X_{j}\beta_{j},$$

where μ is the intercept, ***X****_j_* are design matrices for predictors, ***X****_j_* = **{***_xijk_***}**, and ***β****_j_* are vectors of effects associated with the columns of ***X****_j_*. For the joint analysis model, ***X*_1_** is a design matrix for the effects of breed, ***β*_1_** is the corresponding vector of effects, ***X*_2_** is the matrix with marker genotypes, and ***β*_2_** is the corresponding vector of marker effects. Collecting the above assumptions, we have the following conditional distribution of the data:


$$p(y|\theta )=\prod_{i=1}^{n}N\;\left(y_{i}|\mu +\sum_{j=1}^{J}\sum_{k=1}^{K_{j}}x_{ijk}\beta_{jk},\;\sigma_{ɛ}^{2}w_{i}^{2}\right)$$

where ***θ*** represents the collection of unknowns, including the intercept, regression coefficients (***β****_jk_*) and the residual variance.

This analysis uses Markov Chain Monte Carlo (MCMC) methods to calculate posterior mean estimates of marker effects and variances. The chains included 100,000 iterations with the first 25,000 samples used for burn-in.

The models were evaluated with the Gelman–Rubin diagnostic, which evaluates MCMC convergence by analyzing the difference between multiple Markov chains. The convergence is assessed by comparing the estimated between-chains and within-chain variances for each model parameter. For this, the potential scale reduction factor (***R̂***) was adjusted. The key parameter for this adjustment is the estimated degrees of freedom, d, for a Student-t approximation to the posterior inference based upon the simulations [9],


$${\hat{R}}_{c}=\frac{d+3}{d+1}\hat{R}$$

Large differences between these variances indicate non-convergence (***R̂****_c_*** > 1.1**). When the model did not converge, the number of iterations and burn-in were doubled. Once the effects of the markers were estimated, 95% confidence intervals and posterior probabilities for the markers’ effects were calculated. Marker effects equal to zero reflect no marker's effect.

The SNP associated with growth traits were mapped at 100 kb pairs on either side, because of the average LD (r^2^ = 0.2) previously found in beef cattle [10]. With these SNP windows, genes and quantitative trait loci (QTLs) in the same regions were found. After that, pathway enrichment analyses were conducted using the Database for Annotation, Visualization and Integrated Discovery (DAVID). A p-value <0.05 determined by Fisher’s exact test was set as the criterion for significance. The pathway analyses were carried out using the official symbols of genes and the *Bos taurus* species as reference.

## RESULTS

**Figure 1:**
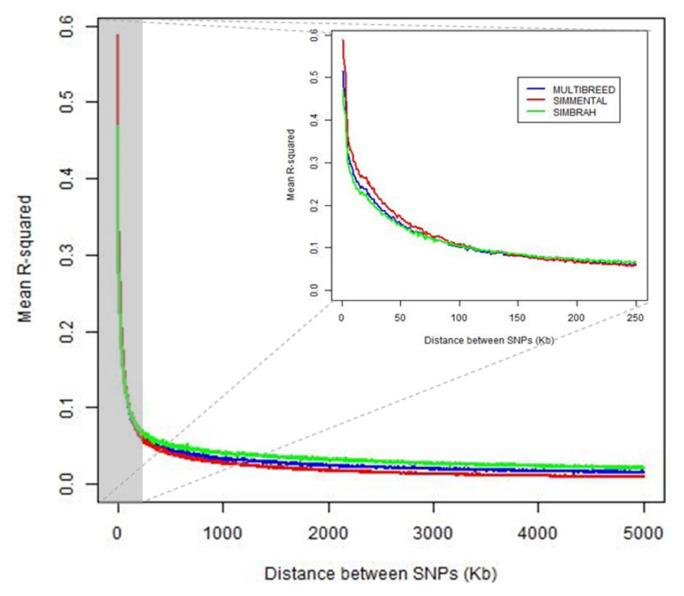
Average coefficients of determination (r^2^) for distance bins of 1 kb for Simmental-, Simbrah- and joint-data analyses plotted against physical distance (kb) between single nucleotide polymorphism.

The intra-chromosomal LD (r^2^) tended to decrease with increasing genomic distance in both breeds and joint data. Simmental showed higher LD than Simbrah in the nearest distances (<100 kb). For larger distances between SNP (>150 kb), Simbrah showed slightly higher average r^2^ than Simmental. For adjacent markers (<1 kb), average r^2^ were 0.515, 0.587, and 0.469 for joint-, Simmental- and Simbrah-data analyses, respectively (Figure 1).

There were differences also in the average r^2^ at a particular marker distance depending on the chromosome. Average r^2^ ranged from 0.019 (BTA26) to 0.057 (BTA14) in the joint analysis, from 0.018 (BTA28) to 0.040 (BTA6) in the Simmental-data analysis, and from 0.023 (BTA26) to 0.068 (BTA14) in the Simbrah-data analysis.

Gelman-Rubin’s shrink factors of all the models converged to 1 as the number of iterations increased, which indicates that five chains converged to each other. To select those markers associated with a trait, posterior probabilities were used. Even though the order of the most associated markers was similar in both GWAS and CWAS, there was a clear difference between the posterior probabilities obtained with both analyses, e.g., in Simmental, the marker BovineHD1300014404 was associated with YW using both CWAS and GWAS; the difference between posterior probabilities was 0.6805 (Table 1). In almost all cases, when a marker was associated with a trait using GWAS, it was also associated with the same trait using CWAS. In contrast, other markers were associated at chromosome-wide level, but were not associated with a trait at genome-wide level.

In both, GWAS and CWAS, SNP were found to be associated with growth traits in the Simmental population (Supplementary Table S1). A total of 23 (16 for GWAS and 7 for CWAS), 22 (11 for GWAS and 11 for CWAS), 28 (21 for GWAS and 7 for CWAS), and 37 (20 for GWAS and 17 for CWAS) SNP were associated with BW, WWD, WWM, and YW, respectively. Additionally, four of the SNP associated with WWM, and WWD were the same and another four SNP shared the same regions (within 100 kb).

In the Simbrah population, a total of 61 (27 for GWAS and 34 for CWAS), 39 (23 for GWAS and 16 for CWAS), 23 (13 for GWAS and 10 for CWAS), and 20 (14 for GWAS and 6 for CWAS) SNP were associated with BW, WWD, WWM, and YW, respectively (Supplementary Table S1); 9 of these SNPs were the same for WWM and WWD. Another four SNP shared the same regions (within 100 kb) for the same traits.

In the joint analysis, 95 (60 for GWAS and 35 for CWAS), 76 (52 for GWAS and 24 for CWAS), 60 (41 for GWAS and 19 for CWAS), and 71 (42 for GWAS and 29 for CWAS) SNP were associated with BW, WWD, WWM and YW, respectively (Supplementary Table S1). Also, 19 and 1 of the SNPs associated with WWM and YW were the same for WWD. Another 3, 1, and 1 SNPs for WWM, BW, and YW shared the same regions (within 100 kb) with SNPs associated with WWD. Within the SNP windows, QTLs were previously associated with other traits, 183, 162, 18, and 302 within the regions associated with BW, WWD, WWM and YW, respectively (Supplementary Table S2). In the same way, a total of 51, 61, 25, and 40 genes were found within these regions (Supplementary Table S2).

In each type of analysis, markers associated with growth traits were found in at least 10 chromosomes, however, there is no clear difference between which chromosome had more associated regions. Displaying all SNP for Simmental, Simbrah and joint model, the SNP for WWD matched 34 and 2 SNP for BW, WWM, and YW, respectively. Also, 3, 2, and 1 SNP windows for BW matched with the SNPs for WWD, WWM, and YW; 5 and 1 SNP windows for WWD matched with the SNPs for WWM and YW; finally, 1 SNP window matched between WWM and YW. Additionally, 14 and 1 of the SNPs associated with WWM and YW were the same for WWD. However, these SNP windows are not close enough to match three or more traits at the same time.

Through the database QTL_ARS_UCD1, 280, 271, 156, and 402 QTLs previously described were found within the SNP windows for BW, WWD, WWM, and YW, respectively (Supplementary Table S2). For all the traits, QTLs associated with conformation, health, meat, carcass, milk, and reproduction traits in cattle were found. Over the database ARS-UCD1.2, 131, 102, 64, and 98 genes were found within the SNP windows for BW, WWD, WWM, and YW, respectively (Supplementary Table S2). Four of these genes were found within SNP windows for WWD and YW.

The posterior probability for gene inclusion is always greater than or equal to the probability that any SNP is included [11]. For this reason, genes within SNP windows were used to search for networks that could be associated with growth traits. Functional enrichment analysis was carried out to identify genes that are over-represented in a large group of genes and may have a connection with the studied phenotypes (Table 2).

## DISCUSSION

The LD estimates at various distances were of the magnitude of those reported by Villa-Angulo et al [12] in several dairy and beef cattle breeds using less dense SNP panels. The difference in the decline of the average r^2^ between Simmental and Simbrah could be an effect of the indicine breeds. It has been observed that indicine breeds had lower r^2^ at short distances and higher r^2^ at longer distances between markers than taurine breeds [13]. Higher LD in taurine breeds has been attributed to smaller effective population size and stronger genetic bottleneck during breed formation [14].

Means of r^2^ were obtained for each chromosome averaged across breeds ranging from 0.019 (BTA26) to 0.057 (BTA14) in the joint model, from 0.018 (BTA28) to 0.040 (BTA6) in Simmental, and from 0.023 (BTA26) to 0.068 (BTA14) in Simbrah. In the present study, the higher LD values detected in some chromosomes in comparison to others can be indicative of the presence of QTLs affecting traits that have been under intense selection in both breeds [6]. A wide variation in autosomal recombination rates can lead to a marked diversity in the pattern of LD in different genomic regions and chromosomes [15]. The causes may have acted differently at specific genomic regions at singular locations among the Simmental and Simbrah populations. The different LD patterns across chromosomes have been assumed that exist, and therefore, it is also expected different genomic inflation factors across chromosomes [16].

The posterior probabilities do not define with clarity the extent of association of an SNP with a trait, however, if we compare with frequentist models, studies have been carried out where it is shown that around half of the published associations with p<5×10^−7^ had posterior probabilities less than 0.5 [17]. The posterior probabilities are being conditioned on the model and the priors, however, while posterior probabilities provide a measure of evidence for hypotheses for the marker effects, it is difficult to judge them separately, as individual model probabilities may be “diluted” as the number of markers grows receiving small probability (both prior and posterior) [11,18]; this could have been what affected the posterior probabilities of the GWAS and CWAS; if this is the case, a lot of useful information could be missing.

The change in posterior probabilities was probably due to the density of the markers used in each of the three analyses, with the GWAS giving lower values compared to those of the CWAS [19]. However, a study applying a mixed linear association model with a leave-one-chromosome-out approach, suggests that even if the genomic inflation factors do not differ a lot between the different SNP densities, genomic inflation factors varied largely across the chromosomes [16]. An explanation might be that there was a different level of association between the SNP on the chromosome and the trait of interest, also, because using the total number of SNPs can result in too conservative thresholds since it violates the assumption of independence between tests [20,21].

For all growth traits, markers were found in regions previously associated with QTLs for production, reproduction, health, and conformation traits (Supplementary Table S2). However, it is of greater interest to focus on the QTLs that are correlated with growth traits.

In the Simmental-data analysis, within the SNP windows, QTL were previously associated with other traits; in total, there were 69, 30, 135, and 71 QTLs within the regions associated with BW, WWD, WWM, and YW, respectively (Supplementary Table S2). A total of 60, 17, 34, and 50 genes were associated with BW, WWD, WWM, and YW, respectively (Supplementary Table S2). For BW, in the associated SNP windows, some QTLs were previously reported with the length of productive life, body depth, net merit, muscle phosphorus and potassium content, shear force, and tenderness score in Holstein, Angus, and Nelore. For WWD, one SNP window on BTA 10 were reported QTLs associated with body weight in Charolais and Gelbvieh cattle. In the case of WWM, inside the SNP windows QTLs were previously associated with average daily gain, body weight gain, body depth, rump width, body weight, carcass weight, fat thickness, hip height, longissimus muscle area, marbling score, metabolic body weight, residual feed intake, withers height, and dry matter intake. For YW, it was found in SNP windows QTLs previously associated with length of productive life, net merit, and dry matter intake in Holstein cattle.

In Simbrah cattle, inside the SNP windows, 28, 79, 3, and 29 QTL were previously associated with other traits within the regions associated with BW, WWD, WWM, and YW, respectively (Supplementary Table S2). Also, a total of 20, 24, 5, and 8 genes were found within these regions (Supplementary Table S2). Within regions associated with BW QTLs were previously found for maturity rate in Brahman cattle and net merit and length of productive life in Holstein cattle. For WWD, in the SNP windows QTLs were previously associated with body weight (birth) in Brangus cattle, weaning weight in Blanco Orejinegro cattle and lean meat yield in Holstein cattle. For WWM, on BTA 24, QTL previously associated with bodyweight and maturity rate were found. For YW, in a SNP window of BTA 14 a QTL previously associated with birth weight in Charolais and Chianina cattle and carcass weight in Hanwoo cattle was found.

From the results of the joint model, several coincidences were found within the regions associated with BW, WWD, and YW, with the databases of QTLs. For BW, in the associated SNP windows, some QTLs previously reported with maturity rate in Brahman cattle, body weight (birth) in Charolais and Chianina cattle, yearling weight in Charolais and Gelbvieh cattle, subcutaneous fat in Hanwoo cattle, body depth, lean meat yield, length of productive life, net merit, PTA type and rump width in Holstein cattle, and dry matter intake and metabolic weight in beef cattle. In the case of WWD inside the SNP windows, QTLs were previously associated with average daily feed intake, carcass weight, fat thickness, marbling score, residual feed intake, and, average daily gain in beef cattle, body weight (yearling) in Charolais, and Chianina cattle, maturity rate in Braham cattle, tenderness score in Angus cattle and, lean meat yield, growth index, average daily gain, length of productive life and net merit in Holstein cattle. For WWM, inside the SNP, windows were reported QTLs associated with muscle sodium content in Angus cattle, maturity rate in Brahman cattle, and dry matter intake in Holstein cattle. For YW it was found in SNP windows QTLs previously associated with marbling score, muscle creatine content, tenderness score in Angus cattle, bodyweight in Charolais, Chianina, and Gelbvieh cattle, carcass weight in Wagyu cattle, dry matter intake, dressed carcass, and body mass in a beef cattle population, and body depth, lean meat yield, length of productive life, net merit and rump width and lactation persistency in Holstein cattle.

The joint analysis of two breeds increases the statistical power to detect more significant SNPs rather than a single analysis. Also, the Simmental and Simbrah analyses give different significant genes. These differences can be associated with the genomic architecture changes with hybridization and subsequent inter-se mating during the formation of a composite breed, this means that alleles at some loci increase in frequency more than others in the newly hybridized population [22]. Additionally, differences were found between the enrichment analyzes, mainly because no similar genes were found between the different association analyses. Furthermore, the more diverse the number of genes, the greater the number of pathways that were found.

In the joint analysis, inside the SNP windows, there are some genes relevant to growth traits (Supplementary Table S2). Among these stand out special AT-rich sequence-binding protein-1 (*SATB1*), prominin mouse-like 1 (*PROM1*), CUB and Sushi multiple domains 1 (*CSMD1*), phosphatidylserine synthase 1 (*PTDSS1*), and ubiquinol-cytochrome c reductase binding protein (*UQCRB*) (candidate genes for BW).

*SATB1* has been associated with the concentration of triiodothyronine, a hormone linked with physiological processes, including growth and development [23]. Also, *SATB1* has shown differences in its expression in fetal adipose tissues, depending on the maternal diet [24]. *PROM1* has been selected as a candidate gene for BW in goats, this is a protein-coding gene, which plays a role in cell differentiation, proliferation, and apoptosis [25]. In Hanwoo cattle, *CSMD1* was more highly expressed in muscle samples from animals with increasing carcass weight in intramuscular fat and eye muscle area [26]. Additionally, *PTDSS1* and *UQCRB* tend to be expressed more highly in muscle with increasing intramuscular fat content [26].

Reactome pathways for WWD in the joint analysis included the metabolism through the regulation of insulin secretion by fatty acids bound to G protein-coupled receptor 40 (*GPR40*) fatty acids augment the glucose-triggered secretion of insulin through two mechanisms: intracellular metabolism and activation of free fatty acid receptor 1 (*FFAR1*).

Also, in the Simmental-data analysis, for BW, a reactome pathway associated with the metabolism of proteins-N-glycan trimming in the endoplasmic reticulum and Calnexin/Calreticulin cycle was identified; in this process, the N-glycan is progressively trimmed off by the three glucoses and some of the mannoses before the protein is transported to the cis-Golgi.

In Simmental cattle, inside the SNP windows, there are some genes relevant to growth traits (Supplementary Table S2). Among these stand out *SATB1* (candidate gene for WWD), proenkephalin (*PENK*), collagen type IV alpha 1 chain (*COL4A1*), short chain dehydrogenase/reductase family 16C member 6 (*SDR16C6*), and cell division cycle 5 like (*CDC5L*) (candidate genes for WWM).

*PENK* has been associated as a candidate gene for growth traits along with RB1 inducible coiled-coil 1 (*RB1CC1*), neuropeptides B and W receptor 1 (*NPBWR1*), and pleiomorphic adenoma gene 1 (*PLAG1*) in Nellore cattle, according to these studies, *PLAG1* seems to be the major gene due to its role in regulating insulin-like growth factors, and *RB1CC1*, *NPBWR1*, and *PENK* are also involved in processes that can contribute to determining the uniformity of growth traits, like YW [27]. *COL4A1* is related to developmental biology, protein digestion, and protein absorption have been indicated as a candidate gene for weaning weight and YW [28]. *SDR16C6* has been associated as a candidate gene for weaning weight and daily weight gain between birth and weaning in Blanco Orejinero cattle [29]. *CDC5L* has been associated as a candidate gene of muscular growth and homeostasis during puberty in conjunction with MYC proto-oncogene (*MYC*), transcription factor 3 (*TCF3*), RUNX family transcription factor 2 (*RUNX2*), activating transcription factor 2 (*ATF2*), and cAMP responsive element binding protein 1 (*CREB1*) [30].

The RNA polymerase pathway was associated with Simmental cattle for BW and take part in the transcription in the genetic information processing. In a study the RNA polymerase pathway was observed downregulated in overfed moderate-energy diet (OVE) cows; also, the gene *POLR2G* (polymerase II gene) and other polymerases III genes were affected. To lower polymerase II gene expression, OVE cows also experienced suppression of the RNA transport pathway. RNA transport allows mRNA transcribed in the nucleus to be processed and translated later in the cytoplasm [31]. This result does not necessarily mean that there was less overall transcription. In eukaryotes, there are 3 distinct RNA polymerases, these transcription complexes are composed of heterogeneous subunits, which can individually affect the transcription complex [32].

The GABAergic synapse pathway was associated with WWD in Simmental cattle. GABA is a neurotransmitter widely distributed in the central nervous system, which is synthesized from glutamate through decarboxylation39 and plays an important role in regulating feeding behavior in the hypothalamus. Other studies have found that this pathway was significantly associated with live weight in Simmental cattle [33]. Additionally, it was suggested that neuronal sensitivity to GABA is related to the control of feeding behavior in ruminant animals [34].

An interesting term that has been found in Simmental cattle for WWD is nicotine and morphine addiction. Both terms are found as significant Kyoto encyclopedia of genes and genomes pathways associated with substance dependence in humans extrapolated to bovines. Other studies have observed these pathways in their analysis [33,35,36]. However, given the origin, all agree that the pathway regulation is ambiguous and requires further validation.

In Simbrah cattle, inside the SNP windows, there are some genes relevant to growth traits (Supplementary Table S2). Among these stand out vacuolar protein sorting 4 homolog B (*VPS4B*) (candidate gene for BW), cadherin 20 (*CDH20*) (candidate gene for WWM), hedgehog acyltransferase (*HHAT*), phosphodiesterase 4B (*PDE4B*), tripartite motif containing 63 (*TRIM63*), high mobility group AT-hook 2 (*HMGA2*) (candidate genes for WWD), and thymocyte selection associated high mobility group box (*TOX*) (candidate gene for YW).

In pigs, *VPS4B* and *CDH20* have been described as a candidate gene that composes the underlying genetic architecture of porcine growth and fatness traits, this gene is crucial for the degradation of membrane receptors, regulation of epidermal growth factor receptors, and insulin receptors [37].

*HHAT* is a gene previously associated with weaning weight in Zebú cattle [38]. *PDE4B* encodes the phosphodiesterase enzyme type 4 that hydrolyses the cyclic adenosine monophosphate, which is related to energy modulation processes in the body, and is linked with lipolysis control, regulating body composition, also this gene has been associated with average daily gain [39]. *TRIM63* is part of the ubiquitin-proteasome system in the main proteolytic pathway in muscle, and the muscle-specific ligases tripartite motif-containing, also there is a supposition that *TRIM63* (MuRF-1) may play a role in the control of protein degradation and probably also contributes to skeletal muscle metabolism [40]. *HMGA2* has been detected for BW in Brangus, the *HMGA* proteins are architectural transcription factors that regulate the transcription of a variety of genes and direct cellular growth, proliferation, and differentiation [41], also the regulation of insulin like growth factor 2 (*IGF2*) by *HMGA2* has been proved to occur directly or through increased expression of *PLAG1* [42].

The *TOX* gene has previously been linked to *PLAG1*, coiled-coil-helix-coiled-coil-helix domain containing 7 (*CHCHD7*), short chain dehydrogenase/reductase family 16C member 5 (*SDR16C5*), *SDR16C6*, *PENK*, family with sequence similarity 110 member B (*FAM110B*), cytochrome P450 family 7 subfamily A member 1 (*CYP7A1*), and syndecan binding protein (*SDCBP*) as candidate genes for carcass weight in Hanwoo cattle. According to them, a denser LD structure was found in and around *TOX* gene rather than a region that surrounds *PLAG1* gene, this result might be due to a multigene effect in which multiple genes in the same QTL region are affecting correlated traits in cattle [43].

In Simbrah, the p53 signaling pathway was significantly associated with BW. This pathway is induced by several stress signals (DNA damage, oxidative stress, and activated oncogenes). The p53 protein is employed as a transcriptional activator of p53-regulated genes. Also, p53 is a gene that has been proposed as a part of a network that influences puberty, making supports the relevance of tumor-related genes for puberty [44]. There is evidence of the correlation of pubertal traits (standardized age at first oestrus and scrotal circumference) with growth characteristics, such as YW and the maternal component of weaning weight and BW [45].

## CONCLUSION

Only 6,265% of the markers associated with growth traits were found using CWAS and GWAS. The associated markers found just in the CWAS could be used for the identification of candidate genes. No significantly associated regions were found between breeds. Although Simbrah is a synthetic breed derived from Simmental, no common regions were found, however, in the joint analysis, some common regions were found.

These regions may be useful in providing insight into growth traits in Simmental and Simbrah cattle with related phenotypic measurements. Also, candidate genes helped identify gene pathways through enrichment analysis. These pathways can help us understand how they are connected to growth traits.

## Figures and Tables

**Table 1:** Single nucleotide polymorphisms (SNP) with the highest posterior probabilities (>0.15) associated with growth traits in both genome-(GWAS) and chromosome-wide analysis (CWAS)

SNP	BW		WWD		WWM		YW	
			
	GWAS	CWAS	GWAS	CWAS	GWAS	CWAS	GWAS	CWAS
Joint analysis								
BovineHD0400015064							0.155	0.660
BovineHD0700022164	0.269	0.552						
BovineHD1100018479			0.448	0.794	0.405	0.907		
BovineHD1200007354			0.188	0.226				
BovineHD1900010762			0.428	0.964				
BovineHD2000005310							0.287	0.914
BovineHD2200002151	0.750	0.860						
BovineHD2900012617	0.664	0.338						
BTB-00218987	0.251	0.482						
BTB-00397480			0.225	0.476				
Simbrah analysis								
ARS-BFGL-NGS-3343	0.288	0.345						
ARS-BFGL-NGS-40907			0.253	0.318	0.152			
ARS-BFGL-NGS-75279			0.210	0.476		0.157		
BovineHD0100005053	0.259	0.750						
BovineHD0200037022				0.272	0.150			
BovineHD0300022871				0.368	0.200	0.687		
BovineHD0400014726			0.171		0.291	0.236		
BovineHD0700030669							0.170	0.694
BovineHD1100018735							0.241	0.903
BovineHD2000005310							0.220	0.503
Simmental analysis								
BovineHD1100018476			0.171	0.675				
BovineHD1100028536							0.338	0.821
BovineHD1300014404							0.151	0.831
BovineHD2100010794			0.153	0.590				
BovineHD2100012099	0.543	0.609						
BovineHD2800009062							0.179	0.471

BW, birth weight; WWD, weaning weight direct; WWM, weaning weight maternal; YW, yearling weight.

**Table 2:** Reactome and KEGG pathways significantly enriched using genes associated with growth traits

Data	Trait	Category	Pathway	Count	p-value	Candidate genes
Joint	WWD	Reactome	Hemostasis-Thrombin signalling through proteinase activated receptors	2	0.02	*GNA11, GNA15*
			Hemostasis-Thromboxane signalling through TP receptor	2	0.0096	*GNA11, GNA15*
			Hemostasis-ADP signalling through P2Y purinoceptor 1	2	0.012	*GNA11, GNA15*
			Hemostasis-Platelet homeostasis-Platelet sensitization by LDL	2	0.011	*GNA11, GNA15*
			Metabolism-Fatty Acids bound to GPR40 regulate insulin secretion	2	0.0096	*GNA11, GNA15*
Simmental	BW	KEGG	RNA polymerase	2	0.035	*POLR2G, POLR3E*
		Reactome	Metabolism of proteins-N-glycan trimming in the endoplasmic reticulum and Calnexin/Calreticulin cycle	2	0.017	*UBXN1, GANAB*
	WWD	KEGG	Nicotine addiction	2	0.022	*GABRR1, GABRR2*
			GABAergic synapse	2	0.045	*GABRR1, GABRR2*
			Morphine addiction	2	0.048	*GABRR1, GABRR2*
Simbrah	BW	KEGG	p53 signaling pathway	2	0.046	*BID, SERPINB5*

KEGG, Kyoto encyclopedia of genes and genomes; *GNA11*,G protein subunit alpha 11; *GNA15*, G protein subunit alpha 15; WWD, weaning weight direct; BW, birth weight; *POLR2G*, RNA polymerase II subunit G; *POLR3E*, RNA polymerase III subunit E; *UBXN1*, UBX domain protein 1; *GANAB*, glucosidase II alpha subunit; *GABRR1*, gamma-aminobutyric acid type A receptor subunit rho1; *GABRR2*, gamma-aminobutyric acid type A receptor subunit rho2; *BID*, BH3 interacting domain death agonist; *SERPINB5*, serpin family B member 5.

## SUPPLEMENTARY MATERIAL

**Supplementary Table S1**. Marker effects ( ) and their posterior probabilities (PP) of SNPs associated with growth traits with the use of genome-wide association (GWA) as well as a chromosome-wide association (CWA) in simmental and simbrah cattle

**Supplementary Table S2**. QTLs and genes previously described within the regions associated with growth traits in simmental and simbrah cattle
