# Supplementary material for: Genome and chromosome wide association studies for growth traits in Simmental and Simbrah cattle
Source: Anim Biosci. 2022 Jun 30;36(1):19–28. doi: 10.5713/ab.21.0517 (PMC9834659; doi:10.5713/ab.21.0517)
Supplement: Supplementary file 1 [file ab-21-0517-suppl1.pdf]

Table S1. Marker effects ($\hat{b}_j$) and their posterior probabilities (PP) of SNPs associated with growth traits with the use of genome-wide association (GWA) as well as a chromosome-wide association (CWA) in simmental and simbrah cattle.

Model	Chr[a]	Pos[b]	SNPs[c]	BW[d]				WWD[e]				WWM[f]				YW[g]			
				GWA		CWA		GWA		CWA		GWA		CWA		GWA		CWA	
				$\hat{b}_i$	PP	$\hat{b}_i$	PP	$\hat{b}_i$	PP	$\hat{b}_i$	PP	$\hat{b}_i$	PP	$\hat{b}_i$	PP	$\hat{b}_i$	PP	$\hat{b}_i$	PP
**Joint**	1	6,952,436	BovineHD0100002043			-0.026	0.129												
**Joint**	1	17,148,366	BovineHD0100005053	0.012	0.066	0.068	0.301												
**Joint**	1	18,258,818	BovineHD0100005339	-0.040	0.240														
**Joint**	1	24,168,476	ARS-BFGL-BAC-6737									0.034	0.043	0.126	0.128				
**Joint**	1	41,455,680	Hapmap23514-BTA-150593					-0.097	0.098			0.083	0.127						
**Joint**	1	49,567,103	BovineHD0100014058			0.014	0.061												
**Joint**	1	49,634,079	BovineHD0100014071			0.013	0.053												
**Joint**	1	50,665,945	BovineHD0100014362	-0.010	0.071	-0.016	0.107												
**Joint**	1	53,241,119	ARS-BFGL-NGS-94761					0.042	0.029	1.713	0.621			-0.112	0.092				
**Joint**	1	54,038,526	BTB-00217542															0.803	0.351
**Joint**	1	58,000,696	ARS-BFGL-NGS-40337					0.139	0.150			-0.037	0.065						
**Joint**	1	78,127,259	BovineHD0100022657			-0.016	0.064												
**Joint**	1	115,897,261	BovineHD0100033028											0.088	0.130				
**Joint**	1	118,821,328	BovineHD0100033867					-0.105	0.106										
**Joint**	1	118,912,426	BovineHD0100033896					-0.116	0.125	-0.623	0.451					-0.089	0.101	-0.466	0.424
**Joint**	1	119,151,202	BovineHD4100000688													-0.045	0.046		
**Joint**	1	131,680,356	BovineHD0100037820					0.058	0.060										
**Joint**	1	147,542,196	BovineHD0100043239									-0.029	0.054						
**Joint**	1	155,615,450	ARS-BFGL-NGS-54653	0.017	0.111	0.078	0.408												
**Joint**	1	157,593,001	BovineHD0100012784													0.044	0.040		
**Joint**	1	157,605,027	BovineHD1100023814													0.034	0.054		
**Joint**	2	6,912,773	BovineHD0200002005					0.048	0.032	0.284	0.200								
**Joint**	2	13,346,155	BTA-20457-no-rs									0.036	0.053						
**Joint**	2	13,367,301	BovineHD4100001011					-0.042	0.034			0.090	0.110	0.337	0.313				
**Joint**	2	28,366,129	BovineHD0200008331							0.190	0.148								
**Joint**	2	30,228,551	BovineHD0200008901							0.238	0.184								
**Joint**	2	57,203,290	BovineHD0200016269			-0.014	0.054												
**Joint**	2	92,668,354	BovineHD0200026560							-0.106	0.100								
**Joint**	2	94,636,270	Hapmap50979-BTA-48487													-0.132	0.152		
**Joint**	2	122,900,223	BovineHD0200035799			-0.013	0.090												
**Joint**	2	133,487,750	BovineHD0200039106									0.054	0.068	0.187	0.169				
**Joint**	3	328,058	BovineHD0300000063									0.045	0.080						

**Joint**	3	15,763,689	BovineHD4100001879			-0.016	0.094												
**Joint**	3	34,241,835	BovineHD0300035533			0.014	0.102												
**Joint**	3	43,071,852	BovineHD0300013160	-0.008	0.038	-0.028	0.102												
**Joint**	3	71,037,098	BovineHD0300020955			-0.013	0.053												
**Joint**	3	73,463,469	BovineHD0300021473	-0.020	0.112	-0.053	0.259												
**Joint**	3	101,605,832	BovineHD0300029255													-0.036	0.045		
**Joint**	4	10,025,067	BovineHD0400003025					-0.037	0.043										
**Joint**	4	34,087,094	BovineHD0400009726	0.016	0.101														
**Joint**	4	39,299,282	BTB-01780264					0.044	0.037	0.361	0.170								
**Joint**	4	45,694,800	BovineHD0400012548					0.095	0.088			-0.203	0.245						
**Joint**	4	48,070,266	BovineHD0400013290					0.107	0.073	1.844	0.838			-0.701	0.473				
**Joint**	4	52,341,148	BovineHD0400014536			-0.151	0.412												
**Joint**	4	52,350,959	BovineHD0400014539	0.007	0.045														
**Joint**	4	53,943,257	BovineHD0400014943													-0.043	0.045		
**Joint**	4	54,668,072	BovineHD0400015064													0.160	0.155	0.964	0.660
**Joint**	4	88,650,637	BovineHD0400024784	0.011	0.074														
**Joint**	4	111,112,243	BovineHD0400032138													0.057	0.074	0.279	0.261
**Joint**	4	112,844,573	BovineHD0400032781					-0.039	0.029			0.047	0.070						
**Joint**	5	4,559,923	ARS-BFGL-NGS-41385							0.829	0.632	-0.029	0.050	-0.246	0.303				
**Joint**	5	8,878,483	BTB-00218987	0.044	0.251	0.097	0.482												
**Joint**	5	28,142,436	BovineHD0500008288													0.126	0.131	0.573	0.458
**Joint**	5	37,375,959	BovineHD0500010721											-0.055	0.052				
**Joint**	5	43,173,154	BovineHD0500012424													-0.037	0.048	-0.274	0.211
**Joint**	5	43,254,214	BTB-01477571													-0.047	0.064	-0.683	0.548
**Joint**	5	46,106,048	BovineHD0500013307			-0.035	0.219												
**Joint**	5	47,114,993	BovineHD0500013676	0.008	0.037	0.056	0.155												
**Joint**	5	47,920,992	BovineHD0500013910					-0.152	0.172			0.084	0.136	0.072	0.096				
**Joint**	5	109,153,222	ARS-BFGL-NGS-16738									0.032	0.054	0.106	0.146				
**Joint**	5	109,639,214	BovineHD0500031760									-0.035	0.063	-0.085	0.116				
**Joint**	5	109,676,735	BovineHD0500031776									-0.040	0.056	-0.063	0.087				
**Joint**	6	206,650	BovineHD0600000030	0.009	0.045														
**Joint**	6	22,449,056	BovineHD0600006577	0.009	0.054														
**Joint**	6	24,537,233	BovineHD4100004318	-0.008	0.057	-0.106	0.543												
**Joint**	6	31,922,816	Hapmap27500-BTC-032985	0.012	0.075														
**Joint**	6	35,575,862	BovineHD4100004463							0.318	0.211								
**Joint**	6	42,770,292	BovineHD0600012095							-0.125	0.103								
**Joint**	6	66,299,180	ARS-BFGL-NGS-72592							-0.194	0.138								
**Joint**	6	73,018,262	BovineHD0600020776													-0.035	0.049		

**Joint**	6	86,762,457	BovineHD0600024201													0.033	0.044		
**Joint**	6	111,363,016	BovineHD0600033010	-0.009	0.062														
**Joint**	6	112,448,301	BovineHD0600033358					-0.042	0.057										
**Joint**	7	7,011,872	BovineHD0700002043													0.043	0.053	0.076	0.058
**Joint**	7	10,456,355	BovineHD0700002995															0.098	0.078
**Joint**	7	10,619,615	BovineHD0700003030													0.034	0.035	0.319	0.250
**Joint**	7	18,446,391	BovineHD0700005476					0.131	0.112										
**Joint**	7	20,673,981	BovineHD0700006039					-0.077	0.069	-0.305	0.205								
**Joint**	7	26,294,867	BovineHD0700007673															0.077	0.093
**Joint**	7	71,945,283	BovineHD0700021817			0.015	0.053												
**Joint**	7	73,180,407	BovineHD0700022161			-0.019	0.110												
**Joint**	7	73,197,856	BovineHD0700022164	-0.046	0.269	-0.112	0.552												
**Joint**	7	73,286,379	BovineHD0700022183	-0.017	0.108	-0.028	0.131												
**Joint**	7	102,495,062	BovineHD0700030660															0.121	0.138
**Joint**	7	102,564,741	BovineHD0700030692													0.059	0.077	0.183	0.186
**Joint**	7	105,857,552	BTB-00115134															0.203	0.133
**Joint**	8	6,189,677	ARS-BFGL-NGS-104101													0.107	0.140		
**Joint**	8	8,989,368	BovineHD0800002828	-0.021	0.142														
**Joint**	8	11,078,911	BovineHD0800003578	-0.015	0.095														
**Joint**	8	60,290,538	ARS-BFGL-NGS-112663					0.043	0.048										
**Joint**	8	67,033,400	ARS-BFGL-NGS-100986			-0.087	0.270												
**Joint**	8	84,579,963	BovineHD0800025526															-0.550	0.201
**Joint**	8	105,641,396	BovineHD0800032157					-0.046	0.042			0.068	0.085	0.116	0.143				
**Joint**	9	11,997,198	ARS-BFGL-NGS-57235													0.044	0.051	0.290	0.302
**Joint**	9	17,288,971	BovineHD0900004742			0.013	0.079												
**Joint**	9	33,787,578	BovineHD0900009384													0.049	0.053	0.306	0.302
**Joint**	9	38,735,093	BovineHD0900010916	0.007	0.050														
**Joint**	9	60,827,498	BTB-00397480					0.259	0.225	0.793	0.476	-0.037	0.063						
**Joint**	9	67,744,737	BovineHD0900019004															-0.183	0.151
**Joint**	9	68,870,505	BovineHD0900019298															-0.141	0.132
**Joint**	9	70,289,809	BovineHD0900019735			0.008	0.053												
**Joint**	9	88,192,486	BovineHD0900025200	-0.008	0.044														
**Joint**	9	94,143,252	BovineHD4100007685			0.007	0.040												
**Joint**	10	8,127,430	BovineHD1000002661													-0.157	0.178		
**Joint**	10	62,797,711	BovineHD1000018187					0.278	0.272										
**Joint**	10	62,841,361	BovineHD1000018212					0.041	0.033										
**Joint**	10	62,884,887	BovineHD1000018235					0.046	0.053										
**Joint**	10	66,652,170	ARS-BFGL-NGS-119477	-0.021	0.138														

**Joint**	10	67,686,585	BovineHD1000019457					0.047	0.048										
**Joint**	10	73,845,211	BovineHD1000021104													-0.056	0.073		
**Joint**	10	86,431,884	BovineHD1000024739	-0.007	0.049														
**Joint**	10	100,924,131	ARS-BFGL-NGS-3343	0.010	0.072														
**Joint**	11	23,992,969	BovineHD1100007232	-0.022	0.155														
**Joint**	11	24,662,197	BovineHD1100007434													0.038	0.028	0.440	0.234
**Joint**	11	35,039,180	BovineHD1100010338					-0.141	0.130	-0.695	0.518	0.055	0.056	0.140	0.156				
**Joint**	11	52,029,467	BovineHD1100015270											0.184	0.177				
**Joint**	11	54,076,261	BovineHD1100015725									-0.042	0.064	-0.278	0.347				
**Joint**	11	64,511,310	BovineHD1100018285													0.066	0.087	0.335	0.337
**Joint**	11	65,438,305	BovineHD1100018470									0.029	0.032						
**Joint**	11	65,466,018	BovineHD1100018476					-0.141	0.123	-0.286	0.175	0.029	0.046						
**Joint**	11	65,476,648	BovineHD1100018479					-0.544	0.448	-1.242	0.794	0.333	0.405	0.941	0.907				
**Joint**	11	88,018,340	ARS-BFGL-BAC-16207					-0.126	0.105										
**Joint**	11	92,059,776	BovineHD1100026733	-0.011	0.076														
**Joint**	11	93,632,997	BovineHD1100027287					0.039	0.031										
**Joint**	11	98,859,170	ARS-BFGL-NGS-1504													0.040	0.040		
**Joint**	11	98,929,180	BovineHD1100028767					0.117	0.119	0.200	0.170								
**Joint**	12	9,614,438	BovineHD1200002648	0.017	0.100	0.075	0.317												
**Joint**	12	14,939,312	BovineHD1200004459					-0.055	0.067										
**Joint**	12	19,910,693	BovineHD1200006022													0.045	0.064		
**Joint**	12	24,453,018	BovineHD1200007354					-0.198	0.188	-0.403	0.226								
**Joint**	12	56,034,163	ARS-BFGL-NGS-104509									-0.031	0.032						
**Joint**	12	56,683,360	BovineHD1200015726					0.046	0.052			-0.104	0.149	-0.198	0.230				
**Joint**	12	56,939,408	BovineHD1200028035					-0.069	0.080	-0.509	0.410								
**Joint**	12	58,592,180	BovineHD1200016189	-0.011	0.065														
**Joint**	12	59,106,604	BovineHD1200016310					-0.101	0.098			0.059	0.096						
**Joint**	12	65,399,981	BovineHD1200018025									-0.054	0.091						
**Joint**	13	1,007,186	BovineHD1300000145	0.020	0.135														
**Joint**	13	1,055,763	BovineHD1300000159	-0.010	0.067														
**Joint**	13	3,792,105	BovineHD4100009863					0.045	0.046			-0.059	0.074						
**Joint**	13	16,380,572	BovineHD1300004626															0.035	0.051
**Joint**	13	73,129,519	BovineHD4100010407									-0.031	0.042						
**Joint**	13	78,533,511	BovineHD1300022916									0.034	0.055						
**Joint**	14	22,518	BovineHD0600000008	0.007	0.044														
**Joint**	14	13,328,209	BovineHD1400004176									0.054	0.070						
**Joint**	14	23,607,944	BovineHD4100011295	-0.010	0.065														
**Joint**	14	28,344,330	BovineHD1400024444	-0.017	0.087														

**Joint**	14	28,438,412	BovineHD1400008725	-0.017	0.085														
**Joint**	14	41,275,153	Hapmap42426-BTA-110900			0.031	0.151												
**Joint**	14	67,912,806	BovineHD1400019721	0.009	0.076	0.071	0.326												
**Joint**	15	2,854,992	BovineHD1500000701									0.041	0.061						
**Joint**	15	4,857,417	Hapmap42143-BTA-23359	0.008	0.047														
**Joint**	15	18,637,239	Hapmap44329-BTA-98197									-0.103	0.132	-0.226	0.221				
**Joint**	15	36,609,370	ARS-BFGL-NGS-108699															0.185	0.210
**Joint**	15	41,077,053	BovineHD1500011406	0.008	0.061														
**Joint**	15	52,246,245	BovineHD1500015330					0.058	0.049	0.637	0.427								
**Joint**	15	55,414,359	BovineHD1500016269					0.044	0.039										
**Joint**	15	65,140,618	ARS-BFGL-NGS-29708													0.056	0.052	1.403	0.742
**Joint**	15	65,148,971	BovineHD1500018910															0.329	0.224
**Joint**	15	68,479,023	BTB-00612553					-0.296	0.233										
**Joint**	15	72,473,829	Hapmap47929-BTA-37615	-0.011	0.080														
**Joint**	15	83,228,283	BovineHD1500024815	0.009	0.062														
**Joint**	16	11,761,343	BovineHD1600003306			-0.095	0.347												
**Joint**	16	14,177,173	BovineHD1600004027	0.014	0.102														
**Joint**	16	69,421,830	BTB-00658688	0.067	0.394														
**Joint**	16	72,830,063	BovineHD1600021349	0.012	0.076														
**Joint**	16	76,408,519	ARS-BFGL-NGS-30589													-0.035	0.031		
**Joint**	17	54,907,144	BovineHD1700016212	0.007	0.031														
**Joint**	17	63,860,049	ARS-BFGL-NGS-94330					0.053	0.066	0.801	0.563								
**Joint**	17	63,950,062	ARS-BFGL-BAC-36611			0.093	0.180												
**Joint**	17	65,618,037	BovineHD1700019693					-0.054	0.047										
**Joint**	18	4,772,831	BovineHD1800001433	0.023	0.144														
**Joint**	18	6,864,571	BTA-42870-no-rs									-0.032	0.062						
**Joint**	18	7,691,780	BTA-44411-no-rs													-0.071	0.076		
**Joint**	18	20,659,854	BTA-42769-no-rs									0.039	0.052						
**Joint**	18	46,276,728	Hapmap50691-BTA-43520	-0.028	0.187														
**Joint**	18	46,311,016	BovineHD1800013676	-0.007	0.044														
**Joint**	19	18,623,450	BovineHD1900005438					0.037	0.051										
**Joint**	19	20,134,133	BovineHD1900005935	-0.010	0.070														
**Joint**	19	36,467,014	BovineHD1900010762					-0.494	0.428	-1.608	0.964								
**Joint**	19	59,245,784	UA-IFASA-8216					0.039	0.032										
**Joint**	19	62,556,282	BovineHD1900018249					0.049	0.056			-0.098	0.129						
**Joint**	20	17,591,241	BovineHD2000005310													-0.286	0.287	-1.276	0.914
**Joint**	20	22,651,571	BTA-95455-no-rs															0.288	0.221
**Joint**	20	26,706,016	BovineHD2000007953															-0.168	0.160

**Joint**	20	48,820,669	BovineHD2000013587	0.015	0.099														
**Joint**	20	60,637,163	BovineHD2000017040									-0.088	0.137						
**Joint**	21	33,958,935	BovineHD2100009841	-0.007	0.058														
**Joint**	21	39,874,490	BovineHD2100011516					-0.141	0.150	-0.983	0.601								
**Joint**	21	46,971,798	ARS-BFGL-NGS-19437	-0.011	0.073														
**Joint**	22	6,983,538	BovineHD2200002151	-0.205	0.750	-0.252	0.860												
**Joint**	22	7,005,226	Hapmap57734-rs29023397	-0.040	0.164														
**Joint**	22	7,615,852	BovineHD2200002310									0.032	0.052						
**Joint**	22	8,374,341	BovineHD2200002533													-0.045	0.045		
**Joint**	22	32,091,984	ARS-BFGL-NGS-68862													0.034	0.048		
**Joint**	22	32,279,858	BovineHD2200009264													0.078	0.086	0.647	0.482
**Joint**	22	49,836,856	ARS-BFGL-NGS-15965													-0.094	0.083		
**Joint**	23	16,132,392	BovineHD2300004168	0.008	0.051														
**Joint**	24	3,673,081	BovineHD2400001090					0.039	0.041										
**Joint**	24	21,855,713	BovineHD2400005992													-0.062	0.065		
**Joint**	24	45,599,385	BovineHD2400012691					0.049	0.056			-0.043	0.055						
**Joint**	24	45,625,444	BovineHD2400012696									-0.047	0.063						
**Joint**	25	24,586,554	BovineHD2500007018									0.037	0.055						
**Joint**	25	27,159,249	BovineHD2500007661													-0.034	0.044		
**Joint**	25	30,331,706	Hapmap27064-BTC-028223					-0.092	0.088										
**Joint**	26	26,877,456	BovineHD2600007223					-0.089	0.103			0.073	0.108						
**Joint**	26	41,389,219	ARS-BFGL-NGS-27004	-0.007	0.047														
**Joint**	27	3,993,708	BovineHD2700000961	-0.010	0.066														
**Joint**	27	4,209,953	BovineHD2700001041	-0.077	0.414														
**Joint**	28	4,045,281	BTB-00974697													0.040	0.051		
**Joint**	28	12,534,832	BovineHD2800003638	-0.010	0.058														
**Joint**	28	26,661,955	ARS-BFGL-NGS-1594					0.050	0.049										
**Joint**	29	39,734,672	Hapmap58798-rs29016922	0.022	0.126	0.166	0.682												
**Joint**	29	41,063,582	BovineHD2900012617	0.158	0.664	0.087	0.338												
**Joint**	29	42,093,690	BovineHD2900012868					-0.087	0.055										
**Joint**	29	44,308,524	BovineHD2900013427													-0.090	0.105		
**Joint**	29	45,254,795	BovineHD2900013642													-0.089	0.109		
**Joint**	29	45,294,962	BovineHD2900013649													-0.050	0.062		
**Joint**	29	46,630,938	BovineHD2900014007													0.048	0.072		
**Joint**	29	46,700,354	ARS-BFGL-NGS-18176													0.057	0.085		
**Simbrah**	1	17,148,366	BovineHD0100005053	0.071	0.259	0.259	0.750												
**Simbrah**	1	24,168,476	ARS-BFGL-BAC-6737					-0.096	0.066	-1.930	0.848	0.117	0.119	1.459	0.900				
**Simbrah**	1	50,665,945	BovineHD0100014362	-0.015	0.056														

**Simbrah**	1	77,723,763	BovineHD0100022553													0.060	0.056	0.218	0.168
**Simbrah**	1	105,630,169	Hapmap27036-BTA-163250			-0.075	0.259												
**Simbrah**	1	105,655,281	BovineHD0100030188			-0.066	0.241												
**Simbrah**	1	113,227,837	Hapmap55381-rs29025399	0.014	0.067														
**Simbrah**	1	130,520,535	BTA-51376-no-rs			-0.041	0.135												
**Simbrah**	1	144,528,438	ARS-BFGL-NGS-76373	-0.020	0.097														
**Simbrah**	2	2,434,950	BovineHD0200000670	0.014	0.071														
**Simbrah**	2	57,203,290	BovineHD0200016269			-0.089	0.189												
**Simbrah**	2	59,904,495	BovineHD0200017150							-0.225	0.146								
**Simbrah**	2	83,004,959	BovineHD0200023844			-0.055	0.190												
**Simbrah**	2	121,510,489	BovineHD0200030201	0.030	0.138														
**Simbrah**	2	126,956,287	BovineHD0200037022					0.195	0.126	0.524	0.272	-0.171	0.150	-0.163	0.134				
**Simbrah**	2	126,959,242	ARS-BFGL-NGS-75279					0.343	0.210	0.974	0.476	-0.166	0.149	-0.185	0.157				
**Simbrah**	3	43,733,400	BovineHD0300013378							0.357	0.228			-0.109	0.111				
**Simbrah**	3	50,662,691	BovineHD0300015419			0.086	0.274												
**Simbrah**	3	52,416,306	ARS-BFGL-NGS-83439					-0.119	0.084	-0.183	0.102			0.114	0.103				
**Simbrah**	3	52,416,878	BovineHD0300015876					-0.084	0.072	-0.212	0.113								
**Simbrah**	3	52,417,560	BovineHD0300015877					-0.135	0.096	-0.219	0.123								
**Simbrah**	3	52,418,852	BovineHD0300015878					-0.129	0.099	-0.239	0.152								
**Simbrah**	3	52,419,897	BovineHD0300015879					-0.104	0.074										
**Simbrah**	3	79,022,233	BovineHD0300022871					-0.106	0.096	-0.573	0.368	0.180	0.200	0.803	0.687				
**Simbrah**	3	95,913,981	BovineHD0300027678			-0.127	0.332												
**Simbrah**	3	97,394,480	BovineHD0300028111											-0.186	0.166				
**Simbrah**	4	5,031,881	BovineHD0400001381			0.056	0.092												
**Simbrah**	4	31,991,755	BovineHD0400009179									-0.046	0.052						
**Simbrah**	4	53,098,825	BovineHD0400014726					-0.226	0.171			0.297	0.291	0.267	0.236				
**Simbrah**	4	54,668,072	BovineHD0400015064													0.080	0.056	1.044	0.569
**Simbrah**	5	6,592,231	BovineHD0500001781			-0.016	0.049												
**Simbrah**	5	12,104,374	ARS-BFGL-NGS-29426											0.329	0.276				
**Simbrah**	5	13,013,473	BovineHD0500003932			0.016	0.074												
**Simbrah**	5	13,022,277	BovineHD0500003937			0.022	0.104												
**Simbrah**	5	19,431,606	ARS-BFGL-NGS-116388					0.069	0.055										
**Simbrah**	5	23,023,551	BovineHD0500006748							1.457	0.725								
**Simbrah**	5	47,920,992	BovineHD0500013910					-0.127	0.107										
**Simbrah**	5	48,404,403	BovineHD0500014027													-0.073	0.069		
**Simbrah**	5	61,740,946	BovineHD0500017392											0.286	0.195				
**Simbrah**	5	64,043,861	BovineHD0500018000			-0.059	0.184												
**Simbrah**	5	103,845,399	BovineHD0500029816			-0.022	0.072												

**Simbrah**	5	109,058,472	ARS-BFGL-NGS-63306	-0.014	0.047														
**Simbrah**	6	26,061,286	BovineHD0600007586			0.084	0.228												
**Simbrah**	6	66,299,180	ARS-BFGL-NGS-72592					-0.118	0.098										
**Simbrah**	6	73,481,332	ARS-BFGL-NGS-101891															0.646	0.323
**Simbrah**	6	100,695,560	BovineHD0600028510	-0.025	0.110	-0.160	0.551												
**Simbrah**	6	112,861,438	BovineHD0600033525									0.046	0.062						
**Simbrah**	7	467,956	BovineHD0700000069	-0.018	0.087														
**Simbrah**	7	53,143,975	BovineHD0700015866			0.057	0.210												
**Simbrah**	7	60,601,468	BovineHD0700018057	0.015	0.072														
**Simbrah**	7	60,660,405	BovineHD0700018070			0.069	0.221												
**Simbrah**	7	60,688,281	BovineHD0700018078			0.074	0.222												
**Simbrah**	7	68,783,389	BovineHD0700020842			-0.094	0.287												
**Simbrah**	7	71,945,283	BovineHD0700021817			0.208	0.595												
**Simbrah**	7	94,642,456	BovineHD0700028320	-0.024	0.105	-0.093	0.309												
**Simbrah**	7	95,124,226	BovineHD0700028470	0.019	0.097														
**Simbrah**	7	96,746,068	BovineHD0700029014	0.047	0.193														
**Simbrah**	7	102,529,075	BovineHD0700030669													0.194	0.170	1.027	0.694
**Simbrah**	7	105,411,287	BovineHD0700031468			0.080	0.252												
**Simbrah**	8	11,078,911	BovineHD0800003578	-0.016	0.076	-0.113	0.370												
**Simbrah**	8	21,572,629	BovineHD0800006587	0.027	0.106	0.080	0.248												
**Simbrah**	8	53,884,363	BovineHD0800016303			-0.044	0.177												
**Simbrah**	8	107,236,795	ARS-BFGL-NGS-41550							-0.415	0.240								
**Simbrah**	9	49,257,679	BTB-02006579					0.070	0.055										
**Simbrah**	9	71,359,980	BovineHD0900020122	-0.025	0.076														
**Simbrah**	10	7,799,488	BTA-118104-no-rs													0.080	0.080		
**Simbrah**	10	64,816,309	ARS-BFGL-NGS-62905			-0.080	0.210												
**Simbrah**	10	100,924,131	ARS-BFGL-NGS-3343	0.073	0.288	0.091	0.345												
**Simbrah**	11	155,522	BovineHD1100000016							0.911	0.470								
**Simbrah**	11	23,992,969	BovineHD1100007232	-0.020	0.103	0.254	0.792												
**Simbrah**	11	47,008,188	ARS-BFGL-NGS-40907					0.373	0.253	0.525	0.318	-0.143	0.152						
**Simbrah**	11	66,281,456	BovineHD1100018735													0.541	0.241	2.662	0.903
**Simbrah**	12	2,618,638	BTB-01103269					0.080	0.056										
**Simbrah**	12	31,449,808	BovineHD1200009267	0.016	0.045														
**Simbrah**	13	37,243,918	BovineHD1300010845													0.113	0.107		
**Simbrah**	13	80,054,531	BovineHD1300023400	-0.028	0.141														
**Simbrah**	14	14,586,053	BovineHD1400004575	-0.028	0.111	-0.136	0.410												
**Simbrah**	14	22,341,542	BovineHD4100011291			-0.085	0.182												
**Simbrah**	14	25,259,499	BovineHD4100011361													0.061	0.046		

**Simbrah**	15	6,233,678	BovineHD1500001629					0.073	0.062			-0.088	0.076						
**Simbrah**	15	72,473,829	Hapmap47929-BTA-37615	-0.052	0.218														
**Simbrah**	16	73,010,856	BovineHD1600021426					0.084	0.064										
**Simbrah**	17	62,581,666	BovineHD1700018671			-0.144	0.428												
**Simbrah**	18	25,185,199	BovineHD1800007788	-0.016	0.071														
**Simbrah**	19	3,880,877	BovineHD1900000960													0.283	0.238		
**Simbrah**	19	21,849,452	BovineHD1900006407	0.016	0.068														
**Simbrah**	20	16,815,306	BovineHD2000005088													0.091	0.070		
**Simbrah**	20	17,591,241	BovineHD2000005310													-0.263	0.220	-0.695	0.503
**Simbrah**	20	35,259,879	BovineHD2000010095					-0.136	0.111	-0.418	0.246								
**Simbrah**	21	59,162,526	ARS-BFGL-NGS-16275	0.034	0.104														
**Simbrah**	21	62,121,957	BovineHD2100018719	-0.026	0.117														
**Simbrah**	21	66,284,836	BovineHD2100019902					0.093	0.076										
**Simbrah**	22	11,083,535	BovineHD2200003294					0.090	0.080										
**Simbrah**	22	33,432,901	BovineHD2200009669					-0.102	0.078			0.086	0.093						
**Simbrah**	23	15,783,127	BovineHD2300004035			0.151	0.499												
**Simbrah**	24	3,673,081	BovineHD2400001090													0.255	0.222		
**Simbrah**	24	4,051,421	BovineHD2400001180									0.047	0.059						
**Simbrah**	24	4,074,679	BovineHD2400001190									0.110	0.122						
**Simbrah**	24	14,964,805	BovineHD2400004198													-0.153	0.152		
**Simbrah**	24	32,576,870	BTB-01623856	-0.015	0.072														
**Simbrah**	24	53,702,809	BovineHD2400015420													-0.203	0.197		
**Simbrah**	24	59,990,135	BovineHD2400017485									0.054	0.051						
**Simbrah**	24	61,732,249	BovineHD2400018109			-0.093	0.276												
**Simbrah**	27	31,269,649	BTB-00966795					-0.099	0.064	-1.434	0.527								
**Simmental**	1	4,615,149	BTB-00002966							0.974	0.573								
**Simmental**	1	30,449,967	ARS-BFGL-NGS-14285															0.652	0.267
**Simmental**	1	39,638,269	BovineHD0100011262									0.089	0.083						
**Simmental**	1	115,487,178	BTA-108007-no-rs									0.056	0.061						
**Simmental**	1	155,461,258	ARS-BFGL-NGS-39036							-0.232	0.117								
**Simmental**	2	20,637,894	ARS-BFGL-NGS-27536			-0.031	0.077												
**Simmental**	2	24,759,987	BTB-00083874			0.023	0.070												
**Simmental**	2	32,163,209	ARS-BFGL-NGS-77689											-0.294	0.273				
**Simmental**	3	8,271,067	BovineHD0300002718											0.250	0.215				
**Simmental**	3	11,445,314	BTA-26230-no-rs													-0.110	0.097		
**Simmental**	3	96,214,989	BovineHD0300027779											-0.277	0.171				
**Simmental**	3	99,860,543	BovineHD0300028809											-0.192	0.123				
**Simmental**	4	10,025,067	BovineHD0400003025					-0.089	0.067										

**Simmental**	4	82,072,066	BovineHD0400022891											-0.109	0.099				
**Simmental**	4	114,805,260	BovineHD0400033491											0.134	0.124				
**Simmental**	5	12,192,068	BovineHD0500003615													0.114	0.096	0.947	0.576
**Simmental**	5	27,758,354	BovineHD0500008183															0.399	0.190
**Simmental**	5	68,696,282	BovineHD0500019361															-0.560	0.361
**Simmental**	5	99,889,487	BovineHD0500028730															0.432	0.232
**Simmental**	5	113,010,737	BovineHD0500032783															0.234	0.155
**Simmental**	6	12,002,585	BovineHD0600003405													-0.087	0.074		
**Simmental**	7	92,877,894	BovineHD0700027753	0.022	0.105														
**Simmental**	8	3,229,083	BovineHD0800001007													0.103	0.079		
**Simmental**	8	6,189,677	ARS-BFGL-NGS-104101													0.079	0.062		
**Simmental**	8	40,320,705	BovineHD0800012081							-0.268	0.140								
**Simmental**	8	78,200,098	BovineHD0800023786									-0.058	0.067						
**Simmental**	8	78,299,132	BovineHD0800023822									-0.108	0.118						
**Simmental**	9	21,845,628	BovineHD0900005901													-0.120	0.073	-3.039	0.967
**Simmental**	9	52,842,486	BovineHD0900014769															-0.860	0.430
**Simmental**	9	60,827,498	BTB-00397480					0.335	0.225			-0.255	0.225						
**Simmental**	10	9,243,642	ARS-BFGL-NGS-5218													0.098	0.068		
**Simmental**	10	45,764,666	BovineHD1000013758	-0.011	0.054														
**Simmental**	10	52,771,028	ARS-BFGL-NGS-14607	0.035	0.148	0.148	0.462												
**Simmental**	10	55,739,309	BovineHD1000016562					0.251	0.141										
**Simmental**	10	62,870,803	Hapmap53237-rs29021167					0.069	0.054										
**Simmental**	10	66,578,924	BovineHD1000019217			0.037	0.133												
**Simmental**	10	76,421,733	BovineHD1000021867					0.079	0.052										
**Simmental**	10	77,297,598	BovineHD1000022170													0.074	0.067		
**Simmental**	11	24,662,197	BovineHD1100007434													0.244	0.113	2.737	0.875
**Simmental**	11	65,014,198	BovineHD1100018385															0.822	0.508
**Simmental**	11	65,438,305	BovineHD1100018470					-0.114	0.092			0.054	0.052						
**Simmental**	11	65,466,018	BovineHD1100018476					-0.244	0.171	-1.345	0.675	0.057	0.062						
**Simmental**	11	65,476,648	BovineHD1100018479					-0.134	0.102	-0.342	0.182	0.088	0.075	0.525	0.423				
**Simmental**	11	67,895,920	BovineHD1100019193			0.045	0.123												
**Simmental**	11	76,334,598	BovineHD1100021866					-0.104	0.077	-1.358	0.683								
**Simmental**	11	80,850,526	BovineHD1100023198													0.112	0.097		
**Simmental**	11	85,981,496	BovineHD1100024691			-0.067	0.209												
**Simmental**	11	98,094,178	BovineHD1100028536													-0.589	0.338	-1.833	0.821
**Simmental**	11	98,222,190	BovineHD1100028591													-0.249	0.177		
**Simmental**	11	104,173,821	BovineHD1100030298															0.607	0.323
**Simmental**	12	84,848,183	BTB-00508549									0.082	0.084						

**Simmental**	13	35,025,679	BovineHD1300010246	-0.017	0.096														
**Simmental**	13	49,369,652	BovineHD1300014404													0.230	0.151	1.800	0.831
**Simmental**	14	16,336,431	BovineHD1400005136															6.288	0.846
**Simmental**	14	23,539,175	BTB-00559128									-0.056	0.064						
**Simmental**	14	23,540,117	BovineHD1400007305									-0.092	0.088						
**Simmental**	14	23,548,867	BovineHD1400007308									-0.064	0.066						
**Simmental**	14	23,550,648	BovineHD1400007309									-0.070	0.066						
**Simmental**	15	31,588,113	BovineHD1500008710									-0.085	0.098						
**Simmental**	15	36,609,370	ARS-BFGL-NGS-108699													0.079	0.059		
**Simmental**	16	62,275,255	BovineHD1600017881															0.981	0.593
**Simmental**	16	73,216,135	BovineHD1600021470	-0.012	0.065														
**Simmental**	17	5,895,955	BTB-01002430							0.419	0.250								
**Simmental**	17	41,889,885	BovineHD1700011883	-0.049	0.245														
**Simmental**	17	63,860,049	ARS-BFGL-NGS-94330							1.126	0.641								
**Simmental**	18	5,082,859	BovineHD1800001572	0.027	0.106														
**Simmental**	18	8,845,340	BovineHD1800003058	0.021	0.094														
**Simmental**	18	60,918,681	ARS-BFGL-NGS-11218	0.024	0.130														
**Simmental**	21	36,357,577	BovineHD2100010794					-0.221	0.153	-1.308	0.590								
**Simmental**	21	41,969,536	BovineHD2100012081	-0.011	0.050														
**Simmental**	21	42,032,839	BovineHD2100012099	-0.133	0.543	-0.161	0.609												
**Simmental**	21	42,043,390	Hapmap42487-BTA-26592	-0.019	0.085														
**Simmental**	22	6,983,538	BovineHD2200002151	-0.011	0.056														
**Simmental**	23	17,791,358	BovineHD2300004479									0.117	0.128						
**Simmental**	25	19,912,086	BovineHD2500005568	-0.047	0.212														
**Simmental**	25	24,586,554	BovineHD2500007018									0.142	0.141						
**Simmental**	25	24,613,223	ARS-BFGL-NGS-97895									0.065	0.066						
**Simmental**	25	30,331,706	Hapmap27064-BTC-028223					-0.262	0.158			0.160	0.136						
**Simmental**	25	40,152,700	BovineHD2500011470													0.070	0.048		
**Simmental**	27	7,754,398	BovineHD2700002124									0.061	0.082						
**Simmental**	27	11,205,123	BovineHD2700003046									0.147	0.158						
**Simmental**	27	18,808,741	BovineHD2700005194							0.339	0.205								
**Simmental**	27	26,428,979	BovineHD2700007194															1.071	0.307
**Simmental**	27	39,714,460	BovineHD2700011482							-0.222	0.150								
**Simmental**	28	12,352,710	BovineHD2800003602													0.092	0.087		
**Simmental**	28	33,405,318	BovineHD2800009010													0.075	0.058		
**Simmental**	28	33,588,312	BovineHD2800009062													0.227	0.179	0.909	0.471
**Simmental**	28	33,608,386	BovineHD2800009066													0.140	0.129		
**Simmental**	28	42,051,760	BovineHD2800011945	-0.016	0.079														

**Simmental**	29	7,859,363	BovineHD2900002232					-0.081	0.086		
**Simmental**	29	41,063,582	BovineHD2900012617	0.016	0.071						
**Simmental**	29	45,254,795	BovineHD2900013642							-0.072	0.069

[a] Chromosome; [b] Position; [c] single nucleotide polymorphism; [d] birth weight; [e] weaning weight direct; [f] weaning weight maternal; [g] yearly weight.
