# Supplementary material for: Genome and chromosome wide association studies for growth traits in Simmental and Simbrah cattle
Source: Anim Biosci. 2022 Jun 30;36(1):19–28. doi: 10.5713/ab.21.0517 (PMC9834659; doi:10.5713/ab.21.0517)
Supplement: Supplementary file 2 [file ab-21-0517-suppl2.pdf]

Table S2. QTLs and genes previously described within the regions associated with growth traits in simmental and simbrah cattle.

Model	Trait	Chr[a]	Pos[b]	SNPs[c]	QTLs[d]	Genes[e]
**Joint**	**BW[f]**	1	155,615,450	ARS-BFGL-NGS-54653	MILK: Milk fat yield (PUBMED_ID=22449276), Milk protein yield (PUBMED_ID=22449276); PRODUCTION: Maturity rate (PUBMED_ID=26445451); REPRODUCTION: Conception rate (PUBMED_ID=31718557)	LOC101908237, LOC112448155, LOC112448186, SATB1
		2	122,900,223	BovineHD0200035799	HEALTH: Bovine respiratory dise (PUBMED_ID=30229962); MILK: Milk kappa-casein perce (PUBMED_ID=27485317), Milk zinc content (PUBMED_ID=25989905)	LOC104971336, LOC112442418, LAPTM5, MATN1
		6	111,363,016	BovineHD0600033010		LOC104968940, TAPT1, PROM1
		8	67,033,400	ARS-BFGL-NGS-100986	HEALTH: Bovine respiratory dise (PUBMED_ID=30229962); MILK: Milk casein percentage (PUBMED_ID=28711251), Milk fat percentage (PUBMED_ID=28711251), Milk fat yield (PUBMED_ID=28711251), Milk protein percentage (PUBMED_ID=28711251), Milk protein-to-fat rat (PUBMED_ID=28711251)	LOC100298923, LOC100848109, LOC784966, LPL
		9	94,143,252	BovineHD4100007685		ZDHHC14
		10	100,924,131	ARS-BFGL-NGS-3343		FOXN3
		11	92,059,776	BovineHD1100026733	HEALTH: Anti-Müllerian hormone (PUBMED_ID=30007805); PRODUCTION: Body weight (birth) (PUBMED_ID=30290764); REPRODUCTION: Conception rate (PUBMED_ID=31718557)	
		12	58,592,180	BovineHD1200016189		TRNAC-GCA_157, LOC784127
		14	22,518	BovineHD0600000008		LOC100299844
		14	67,912,806	BovineHD1400019721		LOC112449543, PTDSS1, MTERF3, UQCRB
		15	83,228,283	BovineHD1500024815	REPRODUCTION: Conception rate (PUBMED_ID=27209127)	MS4A3, LOC112441667, LOC112441666, LOC107133207, MS4A2, MS4A7, MS4A14, MS4A5, MS4A1
		17	63,950,062	ARS-BFGL-BAC-36611	MILK: 305-day milk yield (PUBMED_ID=29521460), Milk fat percentage (PUBMED_ID=29521460), Milk fat yield (PUBMED_ID=29521460), Milk protein percentage (PUBMED_ID=29521460), Milk protein yield (PUBMED_ID=21831322), Milk yield (PUBMED_ID=21831322); REPRODUCTION: Non-return rate (PUBMED_ID=29178833), Sire conception rate (PUBMED_ID=29486732), Sperm motility (PUBMED_ID=27610941)	ACACB, UNG, ALKBH2, USP30, SVOP
		20	48,820,669	BovineHD2000013587		LOC112443046, CDH10
		22	7,005,226	Hapmap57734-rs29023397	MEAT AND CARCASS: Lean meat yield (PUBMED_ID=25273628), Subcutaneous fat (PUBMED_ID=27221246); PRODUCTION: Dry matter intake (PUBMED_ID=23851991), Metabolic body weight (PUBMED_ID=23851991)	LOC107131649, LOC514651, CMTM6, DYNC1LI1

	23	16,132,392	BovineHD2300004168	CONFORMATION: Feet and leg conformati (PUBMED_ID=21831322), Foot angle (PUBMED_ID=21831322), Rear leg placement - re (PUBMED_ID=21831322), Rear leg placement - si (PUBMED_ID=21831322), Stature (PUBMED_ID=21831322), Strength (PUBMED_ID=21831322), Teat length (PUBMED_ID=21831322); HEALTH: Somatic cell score (PUBMED_ID=21831322); MEAT AND CARCASS: Lean meat yield (PUBMED_ID=25273628); MILK: Milk fat percentage (PUBMED_ID=21831322), Milk fat yield (PUBMED_ID=21831322), Milk protein percentage (PUBMED_ID=21831322, 31139206), Milk protein yield (PUBMED_ID=21831322), Milk yield (PUBMED_ID=21831322); PRODUCTION: Body depth (PUBMED_ID=21831322), Net merit (PUBMED_ID=21831322), PTA type (PUBMED_ID=21831322); REPRODUCTION: Calving ease (PUBMED_ID=21831322), Calving ease (maternal) (PUBMED_ID=21831322)	LOC112443875, LOC112443876, LOC112443924, TRERF1
	26	41,389,219	ARS-BFGL-NGS-27004	CONFORMATION: Feet and leg conformati (PUBMED_ID=21831322), Foot angle (PUBMED_ID=21831322), Rear leg placement - re (PUBMED_ID=21831322), Stature (PUBMED_ID=21831322), Strength (PUBMED_ID=21831322), Teat length (PUBMED_ID=21831322), Udder attachment (PUBMED_ID=21831322), Udder depth (PUBMED_ID=21831322), Udder height (PUBMED_ID=21831322); MILK: Milk C16 index (PUBMED_ID=31563305), Milk fat percentage (PUBMED_ID=21831322), Milk fat yield (PUBMED_ID=21831322), Milk palmitoleic acid c (PUBMED_ID=31563305), Milk protein percentage (PUBMED_ID=21831322), Milk protein yield (PUBMED_ID=21831322); PRODUCTION: Body depth (PUBMED_ID=21831322), Body weight (yearling) (PUBMED_ID=19966163), Length of productive li (PUBMED_ID=21831322), Net merit (PUBMED_ID=21831322), PTA type (PUBMED_ID=21831322), Rump width (PUBMED_ID=21831322); REPRODUCTION: Calf size (PUBMED_ID=21183059), Calving ease (PUBMED_ID=21183059, 21831322), Calving ease (maternal) (PUBMED_ID=21831322), Stillbirth (PUBMED_ID=21831322)	FGFR2
	27	4,209,953	BovineHD2700001041	REPRODUCTION: Conception rate (PUBMED_ID=31299913), Reproductive efficiency (PUBMED_ID=26286463)	CSMD1, LOC782601
**WWD**[g]	4	112,844,573	BovineHD0400032781	HEALTH: Tick resistance (PUBMED_ID=28619006); MEAT AND CARCASS: Carcass weight (PUBMED_ID=22479267), Fat thickness at the 12 (PUBMED_ID=22479267), Marbling score (PUBMED_ID=22479267); PRODUCTION: Average daily feed inta (PUBMED_ID=22479267), Average daily gain (PUBMED_ID=22479267), Body weight (yearling) (PUBMED_ID=30290764), Residual feed intake (PUBMED_ID=22479267)	ZBED6CL, LOC112446454, ZNF775, LOC511617, LRRC61, GIMAP8, GIMAP7, RARRES2, REPIN1
	6	112,448,301	BovineHD0600033358	MILK: Milk kappa-casein perce (PUBMED_ID=27485317)	LOC112447132, LOC112447222
	7	20,673,981	BovineHD0700006039	CONFORMATION: Udder depth (PUBMED_ID=21831322); HEALTH: Somatic cell score (PUBMED_ID=21831322); MEAT AND CARCASS: Lean meat yield (PUBMED_ID=25273628); PRODUCTION: Length of productive li (PUBMED_ID=21831322), Net merit (PUBMED_ID=21831322); REPRODUCTION: Age at first calving (PUBMED_ID=25178291), Calving ease (maternal) (PUBMED_ID=21831322), Daughter pregnancy rate (PUBMED_ID=21831322), Stillbirth (maternal) (PUBMED_ID=21831322)	CELF5, TRNAM-CAU_5, LOC112447372, AES, TLE2, S1PR4, TLE6, NCLN, GNA15, GNA11

	11	98,929,180	BovineHD1100028767		SWI5, LOC107132969, TRNAR-UCU_5, LOC112448949, TRNAW-CCA_18, LOC112448915, GOLGA2, MIR199B, MIR3604-2, MIR3154, COQ4, MIR219-1, CERCAM, DNM1, TRUB2, SLC27A4, URM1
	12	59,106,604	BovineHD1200016310	MILK: Milk riboflavin content (PUBMED_ID=25771056)	
	13	3,792,105	BovineHD4100009863	REPRODUCTION: Conception rate (PUBMED_ID=31299913)	LOC112449424, SLX4IP, MKKS
	15	68,479,023	BTB-00612553	CONFORMATION: Rump angle (PUBMED_ID=21831322); HEALTH: Serotonin level (PUBMED_ID=32292413); PRODUCTION: Maturity rate (PUBMED_ID=26445451)	
	17	65,618,037	BovineHD1700019693	REPRODUCTION: Conception rate (PUBMED_ID=28814769), Non-return rate (PUBMED_ID=29178833)	LOC112442055, MYO18B
	19	62,556,282	BovineHD1900018249	MEAT AND CARCASS: Lean meat yield (PUBMED_ID=25273628); MILK: Milk fat yield (PUBMED_ID=21831322), Milk protein yield (PUBMED_ID=21831322, 22449276), Milk yield (PUBMED_ID=21831322); PRODUCTION: Body weight (yearling) (PUBMED_ID=24906442); REPRODUCTION: Non-return rate (PUBMED_ID=24265800)	CEP112, APOH
	24	45,599,385	BovineHD2400012691	HEALTH: Abomasum displacement (PUBMED_ID=23548285)	LOC101903660, EPG5, MIR6523A, MIR6523B, SIGLEC15
	25	30,331,706	Hapmap27064-BTC-028223	MILK: Milk protein yield (PUBMED_ID=22449276), Milking speed (PUBMED_ID=29705414)	AUTS2
	26	26,877,456	BovineHD2600007223	MILK: Milk C14 index (PUBMED_ID=25511820, 26364108, 30243637, 31563305), Milk C16 index (PUBMED_ID=31563305), Milk fat yield (PUBMED_ID=31139206), Milk myristoleic acid c (PUBMED_ID=25511820, 26364108, 31563305), Milk palmitoleic acid c (PUBMED_ID=31563305), Milk protein percentage (PUBMED_ID=22449276); REPRODUCTION: Inseminations per conce (PUBMED_ID=20477799)	
	28	26,661,955	ARS-BFGL-NGS-1594	REPRODUCTION: First service conceptio (PUBMED_ID=32650431), Inseminations per conce (PUBMED_ID=32650431)	PALD1, LRRC20, EIF4EBP2, NODAL
	29	42,093,690	BovineHD2900012868	CONFORMATION: Conformation score (PUBMED_ID=27136002); MEAT AND CARCASS: Tenderness score (PUBMED_ID=29163638); MILK: Milk protein percentage (PUBMED_ID=20630249); PRODUCTION: Average daily gain (PUBMED_ID=27136002), Growth index (PUBMED_ID=27136002); REPRODUCTION: Gestation length (PUBMED_ID=29178833), Interval to first estru (PUBMED_ID=29178833)	C29H11orf95, LOC112444891, PLA2G16, RTN3, ATL3, SPINDOC
**WWM**[h]	1	147,542,196	BovineHD0100043239	MILK: Colostrum albumin conce (PUBMED_ID=33255903)	LOC112448047
	2	133,487,750	BovineHD0200039106	MILK: Milk fat yield (PUBMED_ID=22449276), Milk zinc content (PUBMED_ID=25989905)	LOC112443641, LOC788425, AKR7L, EMC1, MRTO4, UBR4
	5	109,676,735	BovineHD0500031776	CONFORMATION: Conformation score (PUBMED_ID=24341352); MILK: Milk fat percentage (PUBMED_ID=27287773), Milk protein yield (PUBMED_ID=31139206); PRODUCTION: Maturity rate (PUBMED_ID=26445451)	LOC101905166, H1F0, GALR3, MIR658, EIF3L, C5H22orf23, TRIOBP, GCAT, ANKRD54, MICALL1, POLR2F, SOX10
	8	105,641,396	BovineHD0800032157		ASTN2, PAPPA

	13	78,533,511	BovineHD1300022916	MILK: Milk protein percentage (PUBMED_ID=22497459), Milk urea nitrogen yiel (PUBMED_ID=22497459)	LOC101907588, LOC101907513, RIPOR3, PTPN1
	18	20,659,854	BTA-42769-no-rs	PRODUCTION: Dry matter intake (PUBMED_ID=23031337)	
	20	60,637,163	BovineHD2000017040	MEAT AND CARCASS: Muscle sodium content (PUBMED_ID=29163638); REPRODUCTION: First service conceptio (PUBMED_ID=32650431), Inseminations per conce (PUBMED_ID=32650431)	
**YW**[i]	1	157,605,027	BovineHD1100023814		LOC107131289, PRDM9, ZNF596
	2	94,636,270	Hapmap50979-BTA-48487	MEAT AND CARCASS: Muscle creatine content (PUBMED_ID=29163638); MILK: Milk kappa-casein perce (PUBMED_ID=27485317), Milk protein percentage (PUBMED_ID=25148050); PRODUCTION: Body weight (birth) (PUBMED_ID=19966163), Body weight gain (PUBMED_ID=19966163), Dry matter intake (PUBMED_ID=23851991), Metabolic body weight (PUBMED_ID=23851991)	LOC112442953, ZDBF2, GPR1, ADAM23
	3	101,605,832	BovineHD0300029255	MILK: Milk kappa-casein perce (PUBMED_ID=27485317), Milk unglycosylated kap (PUBMED_ID=27485317)	RNF220, ERI3
	4	111,112,243	BovineHD0400032138	MEAT AND CARCASS: Yield grade (PUBMED_ID=22394233); MILK: Milk protein percentage (PUBMED_ID=27506634); PRODUCTION: Body weight (yearling) (PUBMED_ID=19966163)	CNTNAP2
	6	86,762,457	BovineHD0600024201	CONFORMATION: Angularity (PUBMED_ID=29115939), Feet and leg conformati (PUBMED_ID=21831322), Foot angle (PUBMED_ID=21831322), Rear leg placement - si (PUBMED_ID=21831322), Teat placement - front (PUBMED_ID=21831322), Udder attachment (PUBMED_ID=21831322), Udder depth (PUBMED_ID=21831322); HEALTH: Bovine tuberculosis sus (PUBMED_ID=26960806), Clinical mastitis (PUBMED_ID=23647142, 27760518), Ketosis (PUBMED_ID=31311492), Somatic cell count (PUBMED_ID=27534682, 21831322, 31139206); MILK: Cheese fat recovery (PUBMED_ID=27889122), Milk alpha-S2-casein pe (PUBMED_ID=27485317), Milk fat yield (PUBMED_ID=31139206), Milk glycosylated kappa (PUBMED_ID=27485317), Milk kappa-casein perce (PUBMED_ID=27485317), Milk phosphorylated alp (PUBMED_ID=27485317), Milk protein percentage (PUBMED_ID=20630249, 22449276, 27287773, 31139206), Milk protein yield (PUBMED_ID=27760518, 28358110, 29751743, 29921979, 30696404, 31139206), Milk rennet coagulation (PUBMED_ID=26947304), Milk unglycosylated kap (PUBMED_ID=27485317), Milk yield (PUBMED_ID=25084281, 25151887, 31139206), Time to curd firmness (PUBMED_ID=26947304); PRODUCTION: Lactation persistency (PUBMED_ID=27889128), Length of productive li (PUBMED_ID=21831322, 27889128), Net merit (PUBMED_ID=21831322); REPRODUCTION: Calving ease (PUBMED_ID=21831322), Calving ease (maternal) (PUBMED_ID=21831322), Conception rate (PUBMED_ID=31139206), Daughter pregnancy rate (PUBMED_ID=27209127, 31139206), Interval to first estru (PUBMED_ID=20477799, 28814769), Stillbirth (maternal) (PUBMED_ID=21831322)	SLC4A4
	7	105,857,552	BTB-00115134	MILK: Milk protein yield (PUBMED_ID=22449276), Milk tridecylic acid co (PUBMED_ID=27506634)	
	8	84,579,963	BovineHD0800025526	MEAT AND CARCASS: Carcass weight (PUBMED_ID=22607022); MILK: Milking speed (PUBMED_ID=29705414)	LOC112447923, CARD19, WNK2, NINJ1
	9	68,870,505	BovineHD0900019298		SMLR1, EPB41L2
	12	19,910,693	BovineHD1200006022	HEALTH: Bovine tuberculosis sus (PUBMED_ID=30763354); MEAT AND CARCASS: Lean meat yield (PUBMED_ID=25273628)	LOC112449043

		15	65,148,971	BovineHD1500018910	MILK: Milk C14 index (PUBMED_ID=31563305), Milk capric acid conten (PUBMED_ID=30841852, 31563305), Milk fat percentage (PUBMED_ID=31139206), Milk fat yield (PUBMED_ID=29751743, 30696404, 31139206), Milk lauric acid conten (PUBMED_ID=31563305); PRODUCTION: Body weight (yearling) (PUBMED_ID=30290764)	ELF5, EHF
		16	76,408,519	ARS-BFGL-NGS-30589	CONFORMATION: Dairy form (PUBMED_ID=21831322), Teat length (PUBMED_ID=21831322); HEALTH: Somatic cell score (PUBMED_ID=21831322); MEAT AND CARCASS: Marbling score (PUBMED_ID=29163638), Tenderness score (PUBMED_ID=29163638); PRODUCTION: Length of productive li (PUBMED_ID=21831322); REPRODUCTION: Daughter pregnancy rate (PUBMED_ID=21831322)	LOC112441872, DENND1B, MIR2284N, CRB1
		18	7,691,780	BTA-44411-no-rs		LOC101902820, LOC112442253, LOC112442481, PKD1L2, CMC2, CENPN, C18H16orf46, GCSH, ATMIN
		20	26,706,016	BovineHD2000007953	PRODUCTION: Body weight (yearling) (PUBMED_ID=19966163), Length of productive li (PUBMED_ID=27889128)	
		22	49,836,856	ARS-BFGL-NGS-15965	CONFORMATION: Hoof and leg disorders (PUBMED_ID=27344389); HEALTH: Ketosis (PUBMED_ID=31311492)	LOC104975568, HEMK1, C22H3orf18, CACNA2D2, MAPKAPK3, CISH
		29	46,700,354	ARS-BFGL-NGS-18176		LOC112444901
**Simbrah**	**BW**	3	95,913,981	BovineHD0300027678		LOC112446108, FAF1, DMRTA2
		5	109,058,472	ARS-BFGL-NGS-63306	CONFORMATION: Foot angle (PUBMED_ID=18565942)	LOC112446914, TRNAE-UUC_32, LOC104972584, CECR2, BCL2L13, ATP6V1E1, BID
		13	80,054,531	BovineHD1300023400	MILK: Milk glycerophosphochol (PUBMED_ID=25670729)	
		19	21,849,452	BovineHD1900006407	REPRODUCTION: Conception rate (PUBMED_ID=31299913), First service conceptio (PUBMED_ID=31299913)	ABR, TIMM22, NXN
		21	62,121,957	BovineHD2100018719	MILK: Milk conjugated linolei (PUBMED_ID=24909189); REPRODUCTION: Calving ease (PUBMED_ID=29178833)	LOC112443345
		24	61,732,249	BovineHD2400018109	CONFORMATION: Feet and leg conformati (PUBMED_ID=21831322), Foot angle (PUBMED_ID=21831322), Rear leg placement - re (PUBMED_ID=21831322), Rear leg placement - si (PUBMED_ID=21831322), Rump angle (PUBMED_ID=21831322), Stature (PUBMED_ID=21831322), Strength (PUBMED_ID=21831322), Teat length (PUBMED_ID=21831322), Udder depth (PUBMED_ID=21831322); HEALTH: Somatic cell score (PUBMED_ID=21831322); MILK: Milk fat percentage (PUBMED_ID=21831322), Milk fat yield (PUBMED_ID=21831322), Milk potassium content (PUBMED_ID=25989905), Milk protein percentage (PUBMED_ID=21831322), Milk protein yield (PUBMED_ID=21831322), Milk yield (PUBMED_ID=21831322); PRODUCTION: Length of productive li (PUBMED_ID=21831322), Maturity rate (PUBMED_ID=26445451), Net merit (PUBMED_ID=21831322); REPRODUCTION: Calving ease (PUBMED_ID=21831322), Calving ease (maternal) (PUBMED_ID=21831322), Stillbirth (PUBMED_ID=21831322)	LOC112444232, LOC107131790, SERPINB12, VPS4B, KDSR, SERPINB5

	**WWD**	2	126,959,242	ARS-BFGL-NGS-75279	CONFORMATION: Rump angle (PUBMED_ID=31633203); MILK: Milk fat yield (PUBMED_ID=31139206), Milk lactose content (PUBMED_ID=29246110), Milk tetracosanoic acid (PUBMED_ID=27506634), Milk zinc content (PUBMED_ID=25989905); REPRODUCTION: Conception rate (PUBMED_ID=31299913)	LOC112443410, LOC112443411, LOC107131877, CATSPER4, CEP85, CNKSR1, ZNF593, FAM110D, PDIK1L, TRIM63, SLC30A2, EXTL1
		3	79,022,233	BovineHD0300022871	MILK: Milk yield (PUBMED_ID=22449276)	LOC101902048, PDE4B
		5	47,920,992	BovineHD0500013910	CONFORMATION: Stature (PUBMED_ID=21212230, 29459679), Teat thickness (PUBMED_ID=28727049), Udder cleft (PUBMED_ID=28727049); PRODUCTION: Body weight (birth) (PUBMED_ID=24906442); REPRODUCTION: Gestation length (PUBMED_ID=22034999), Inhibin level (PUBMED_ID=23785023), Interval to first estru (PUBMED_ID=22100599), Pregnancy rate (PUBMED_ID=26020876)	HMGA2, MIR763
		11	155,522	BovineHD1100000016		LOC112448861
		16	73,010,856	BovineHD1600021426	PRODUCTION: Body weight (weaning) (PUBMED_ID=25158260), Lactation persistency (PUBMED_ID=20412936)	LOC112441917, LOC112441868, HHAT, SERTAD4
		20	35,259,879	BovineHD2000010095	HEALTH: Abomasum displacement (PUBMED_ID=23548285), Respiratory rate (PUBMED_ID=26198991); MEAT AND CARCASS: Lean meat yield (PUBMED_ID=25273628); MILK: Milk fat content (PUBMED_ID=30459810), Milk fat percentage (PUBMED_ID=20630249, 31139206), Milk fat yield (PUBMED_ID=22449276, 30459810), Milk protein percentage (PUBMED_ID=20630249, 21048968, 22449276, 27287773, 28377602, 31139206), Milk protein yield (PUBMED_ID=22449276), Milk yield (PUBMED_ID=31139206); REPRODUCTION: Calving to conception i (PUBMED_ID=28259397), Inseminations per conce (PUBMED_ID=28259397)	FYB1
		21	66,284,836	BovineHD2100019902	HEALTH: Immunoglobulin G level (PUBMED_ID=30241501)	LOC112443172
		22	33,432,901	BovineHD2200009669	HEALTH: Somatic cell score (PUBMED_ID=25288516)	FAM19A1
	**WWM**	6	112,861,438	BovineHD0600033525	REPRODUCTION: Retained placenta (PUBMED_ID=31931710)	QDPR, CLRN2
		24	59,990,135	BovineHD2400017485	PRODUCTION: Body weight (yearling) (PUBMED_ID=19966163), Maturity rate (PUBMED_ID=26445451)	LOC112444177, LOC101902622, CDH20
	**YW**	14	25,259,499	BovineHD4100011361	HEALTH: Insulin-like growth fac (PUBMED_ID=22811567); MEAT AND CARCASS: Carcass weight (PUBMED_ID=24116007, 25164077, 26104396, 27221246); MILK: Milking speed (PUBMED_ID=29705414); PRODUCTION: Body weight (birth) (PUBMED_ID=30290764); REPRODUCTION: Age at puberty (PUBMED_ID=22100599), Interval to first estru (PUBMED_ID=22100599), Scrotal circumference (PUBMED_ID=22811567)	TRNAC-GCA_175, TOX
		19	3,880,877	BovineHD1900000960	HEALTH: Bovine tuberculosis sus (PUBMED_ID=30763354)	
		24	53,702,809	BovineHD2400015420		MBD2, LOC104975792, LOC112441476, STARD6, POLI, C24H18orf54
**Simmental**	**BW**	16	73,216,135	BovineHD1600021470	MEAT AND CARCASS: Muscle phosphorus conte (PUBMED_ID=29163638), Muscle potassium conten (PUBMED_ID=29163638)	
		16	73,216,135	BovineHD1600021470		SYT14

	18	60,918,681	ARS-BFGL-NGS-11218	CONFORMATION: Foot angle (PUBMED_ID=21831322), Rear leg placement - si (PUBMED_ID=21831322), Strength (PUBMED_ID=21831322); HEALTH: Bovine tuberculosis sus (PUBMED_ID=26960806), Somatic cell score (PUBMED_ID=21831322); MILK: Milk fat percentage (PUBMED_ID=21831322), Milk fat yield (PUBMED_ID=21831322), Milk protein percentage (PUBMED_ID=21831322), Milk protein yield (PUBMED_ID=21831322), Milk tridecylic acid co (PUBMED_ID=27506634); PRODUCTION: Body depth (PUBMED_ID=21831322), Length of productive li (PUBMED_ID=27889128), Net merit (PUBMED_ID=21831322); REPRODUCTION: Calving ease (PUBMED_ID=21831322, 28109604), Conception rate (PUBMED_ID=28814769), Interval from first to (PUBMED_ID=28814769), Stayability (PUBMED_ID=29471369), Stillbirth (PUBMED_ID=21831322, 28109604)	
	18	60,918,681	ARS-BFGL-NGS-11218		LOC532048, LOC504704, LOC615600, LOC515600, LOC506868, MGC139164, MIR371, MGC157082, NLRP12
	25	19,912,086	BovineHD2500005568	HEALTH: Anti-Müllerian hormone (PUBMED_ID=29729909); MILK: Average daily milk yiel (PUBMED_ID=28857209), Milk tridecylic acid co (PUBMED_ID=27506634)	LOC112444373, SDR42E2, LOC112444300, TRNAL-UAG_3, TRNAL-AAG_9, VWA3A, POLR3E, EEF2K, CDR2
	28	42,051,760	BovineHD2800011945	MEAT AND CARCASS: Shear force (PUBMED_ID=28727016)	LOC112444747, LOC112444750, LOC112444720, LOC101907562, ANXA8L1, ANTXRL
	29	41,063,582	BovineHD2900012617	MEAT AND CARCASS: Tenderness score (PUBMED_ID=29163638); MILK: 305-day milk yield (PUBMED_ID=ISU0100), Milk fat percentage (PUBMED_ID=20630249), Milk protein percentage (PUBMED_ID=20630249, 27485317, 32998688), Milk yield (PUBMED_ID=20630249); REPRODUCTION: Interval to first estru (PUBMED_ID=29178833)	LOC112444961, LBHD1, LOC112444859, LOC112444936, LOC104976274, LOC112444959, LOC112444957, LOC112444955, LOC112444950, LOC112444960, LOC112444952, LOC112444953, LOC112444954, LOC112444948, C29H11orf98, ZBTB3, UQCC3, HNRNPUL2, TTC9C, B3GAT3, GANAB, INTS5, CSKMT, UBXN1, LRRN4CL, BSCL2, GNG3, POLR2G, TAF6L, TMEM179B, TMEM223, NXF1, STX5, WDR74, SLC3A2
**WWD**	1	155,461,258	ARS-BFGL-NGS-39036	MILK: Milk fat yield (PUBMED_ID=22449276), Milk protein yield (PUBMED_ID=22449276)	LOC112448269, LOC101908237, LOC112448155, SATB1
	9	60,827,498	BTB-00397480	MILK: Milk alpha-S2-casein pe (PUBMED_ID=27485317), Milk phosphorylated alp (PUBMED_ID=27485317); REPRODUCTION: Inseminations per conce (PUBMED_ID=24428918)	LOC101903075, LOC100847428, LOC112448207, ANKRD6, MIR2903, GABRR1, RRAGD, UBE2J1, GABRR2
	10	76,421,733	BovineHD1000021867	MILK: Milk glycosylated kappa (PUBMED_ID=27485317); PRODUCTION: Body weight (yearling) (PUBMED_ID=19966163), Body weight gain (PUBMED_ID=19966163); REPRODUCTION: Conception rate (PUBMED_ID=31718557), Inseminations per conce (PUBMED_ID=31718557)	LOC112448595, LOC100297513, SYNE2, ESR2
	27	39,714,460	BovineHD2700011482	MILK: Milk fat percentage (PUBMED_ID=29284405)	

**WWM**	3	99,860,543	BovineHD0300028809	MILK: Milk kappa-casein perce (PUBMED_ID=27485317), Milk unglycosylated kap (PUBMED_ID=27485317)	LOC104971814, LOC101902049, LOC112446064, NSUN4, UQCRH, LRRC41, LURAP1, POMGNT1, PIK3R3, RAD54L, TSPAN1
	4	114,805,260	BovineHD0400033491	HEALTH: Somatic cell score (PUBMED_ID=25288516); MEAT AND CARCASS: Carcass weight (PUBMED_ID=30290764); PRODUCTION: Average daily gain (PUBMED_ID=24730749), Body weight gain (PUBMED_ID=19966163)	CCT8L2, KMT2C
	11	65,476,648	BovineHD1100018479	MILK: Milk fat percentage (PUBMED_ID=25511820)	LOC100139826
	12	84,848,183	BTB-00508549	CONFORMATION: Feet and leg conformati (PUBMED_ID=21831322), Foot angle (PUBMED_ID=21831322), Rear leg placement - re (PUBMED_ID=21831322), Stature (PUBMED_ID=21831322), Strength (PUBMED_ID=21831322), Udder attachment (PUBMED_ID=21831322), Udder cleft (PUBMED_ID=21831322), Udder depth (PUBMED_ID=21831322), Udder height (PUBMED_ID=21831322); PRODUCTION: Body depth (PUBMED_ID=21831322), PTA type (PUBMED_ID=21831322), Rump width (PUBMED_ID=21831322)	COL4A1
	14	23,550,648	BovineHD1400007309	CONFORMATION: Stature (PUBMED_ID=21212230); HEALTH: Insulin-like growth fac (PUBMED_ID=22811567, 23785023); MEAT AND CARCASS: Carcass weight (PUBMED_ID=22497335, 22607022, 27112906), Fat thickness at the 12 (PUBMED_ID=22497335), Longissimus muscle area (PUBMED_ID=22497335), Marbling score (PUBMED_ID=22497335); PRODUCTION: Average daily gain (PUBMED_ID=22497335), Body weight (PUBMED_ID=26641032), Body weight (birth) (PUBMED_ID=19966163), Body weight (weaning) (PUBMED_ID=19966163), Body weight (yearling) (PUBMED_ID=19966163), Body weight gain (PUBMED_ID=19966163), Hip height (PUBMED_ID=31179577), Metabolic body weight (PUBMED_ID=28521758, 31931702), Residual feed intake (PUBMED_ID=22497335), Withers height (PUBMED_ID=31179577); REPRODUCTION: Age at puberty (PUBMED_ID=22100599, 26641032, 30997484), Calving ease (PUBMED_ID=24906442), Interval to first estru (PUBMED_ID=22100599, 26641032), Scrotal circumference (PUBMED_ID=22811567), Sexual precocity (PUBMED_ID=30053002)	SDR16C6, PENK
	15	31,588,113	BovineHD1500008710	PRODUCTION: Body weight (yearling) (PUBMED_ID=19966163)	LOC785951, GRIK4, TBCEL
	23	17,791,358	BovineHD2300004479	MILK: Milk protein percentage (PUBMED_ID=31139206), Milk yield (PUBMED_ID=28358110, 29921979); PRODUCTION: Body weight gain (PUBMED_ID=19966163), Dry matter intake (PUBMED_ID=24183684)	LOC101905117, MYMX, LOC101905365, SPATS1, TCTE1, CAPN11, HSP90AB1, TMEM151B, SLC29A1, SLC35B2, NFKBIE, AARS2, CDC5L
	27	11,205,123	BovineHD2700003046	PRODUCTION: Average daily gain (PUBMED_ID=22497295), Lactation persistency (PUBMED_ID=20412936)	
	29	7,859,363	BovineHD2900002232	REPRODUCTION: Conception rate (PUBMED_ID=31718557), Inseminations per conce (PUBMED_ID=31718557)	LOC112444877

**YW**	5	113,010,737	BovineHD0500032783	CONFORMATION: Dairy form (PUBMED_ID=21831322), Feet and leg conformati (PUBMED_ID=21831322), Foot angle (PUBMED_ID=21831322), Rear leg placement - re (PUBMED_ID=21831322), Rear leg placement - si (PUBMED_ID=21831322), Strength (PUBMED_ID=21831322), Teat length (PUBMED_ID=21831322); HEALTH: Somatic cell score (PUBMED_ID=21831322); MILK: Milk fat percentage (PUBMED_ID=21831322), Milk fat yield (PUBMED_ID=21831322), Milk protein percentage (PUBMED_ID=21831322), Milk protein yield (PUBMED_ID=21831322); PRODUCTION: Length of productive li (PUBMED_ID=21831322), Net merit (PUBMED_ID=21831322); REPRODUCTION: Calving ease (PUBMED_ID=21831322), Calving ease (maternal) (PUBMED_ID=21831322), Daughter pregnancy rate (PUBMED_ID=21831322), Inseminations per conce (PUBMED_ID=32271764), Interval to first estru (PUBMED_ID=22100599), Stillbirth (PUBMED_ID=21831322)	LOC535121, LOC785804, MGC127055, CYP2D14, MIR2442, CENPM, 37865, WBP2NL, NAGA, PHETA2, SMDT1, NDUFA6, TCF20
	10	77,297,598	BovineHD1000022170		LOC112448644, MAX
	11	104,173,821	BovineHD1100030298	MILK: 305-day milk yield (PUBMED_ID=ISU0100), Cheese protein recovery (PUBMED_ID=27889122), Milk beta-lactoglobulin (PUBMED_ID=27485317, 29571285), Milk butyric acid conte (PUBMED_ID=29391528), Milk kappa-casein perce (PUBMED_ID=27485317)	LOC101902839, LOC101902895, LOC107132967, LOC112448923, LOC112448928, LOC100848307, LOC112448857, LOC112448956, LOC112448907, LOC112448908, LOC112448904, LOC112448903, LOC112448905, EGFL7, FAM69B, LOC789606, LOC100139115, SURF2, MIR126, SURF4, AGPAT2, ABO, SURF6, MED22, RPL7A
	13	49,369,652	BovineHD1300014404	MILK: Milk protein percentage (PUBMED_ID=31139206), Milk yield (PUBMED_ID=22449276)	LOC112449233
	25	40,152,700	BovineHD2500011470	PRODUCTION: Dry matter intake (PUBMED_ID=24183684)	SDK1, MIR2390
	27	26,428,979	BovineHD2700007194	HEALTH: Bovine tuberculosis sus (PUBMED_ID=31480266)	LOC101906081, LOC112441503, LOC112444672, DCTN6, RBPMS
	28	33,608,386	BovineHD2800009066	MILK: Time to curd firmness (PUBMED_ID=26947304)	DLG5, POLR3A

[a] Chromosome; [b] Position; [c] single nucleotide polymorphism; [d] QTLs found in the NAGRP CattleQTLdb database (Available online: https://www.animalgenome.org/cgi-bin/QTLdb/BT/index.); [e] genes found in the assembly ARS-
